# A new Hoxb8FlpO mouse line for intersectional approaches to dissect developmentally defined adult sensorimotor circuits

**DOI:** 10.3389/fnmol.2023.1176823

**Published:** 2023-08-01

**Authors:** Manon Bohic, Aman Upadhyay, Jaclyn T. Eisdorfer, Jessica Keating, Rhiana C. Simon, Brandy A. Briones, Chloe Azadegan, Hannah D. Nacht, Olisemeka Oputa, Alana M. Martinez, Bridget N. Bethell, Mark A. Gradwell, Peter Romanienko, Matt S. Ramer, Garret D. Stuber, Victoria E. Abraira

**Affiliations:** ^1^Cell Biology and Neuroscience Department, Rutgers University, The State University of New Jersey, Piscataway, NJ, United States; ^2^W.M. Keck Center for Collaborative Neuroscience, Rutgers University, The State University of New Jersey, Piscataway, NJ, United States; ^3^Neuroscience PhD Program at Rutgers Robert Wood Johnson Medical School, Piscataway, NJ, United States; ^4^School of Medicine, Oregon Health and Science University, Portland, OR, United States; ^5^M.D./PhD Program in Neuroscience, School of Medicine, Oregon Health and Science University, Portland, OR, United States; ^6^Center for the Neurobiology of Addiction, Pain, and Emotion, Department of Anesthesiology and Pain Medicine, Department of Pharmacology, University of Washington, Seattle, WA, United States; ^7^International Collaboration on Repair Discoveries and Department of Zoology, The University of British Columbia, Vancouver, BC, Canada; ^8^Genome Editing Shared Resource, Rutgers Cancer Institute of New Jersey, New Brunswick, NJ, United States

**Keywords:** neuroscience, spinal cord, sensorimotor system, dorsal root ganglion (DRG), genetic tools

## Abstract

Improvements in the speed and cost of expression profiling of neuronal tissues offer an unprecedented opportunity to define ever finer subgroups of neurons for functional studies. In the spinal cord, single cell RNA sequencing studies support decades of work on spinal cord lineage studies, offering a unique opportunity to probe adult function based on developmental lineage. While Cre/Flp recombinase intersectional strategies remain a powerful tool to manipulate spinal neurons, the field lacks genetic tools and strategies to restrict manipulations to the adult mouse spinal cord at the speed at which new tools develop. This study establishes a new workflow for intersectional mouse-viral strategies to dissect adult spinal function based on developmental lineages in a modular fashion. To restrict manipulations to the spinal cord, we generate a brain-sparing *Hoxb8^FlpO^* mouse line restricting Flp recombinase expression to caudal tissue. Recapitulating endogenous *Hoxb8* gene expression, Flp-dependent reporter expression is present in the caudal embryo starting day 9.5. This expression restricts Flp activity in the adult to the caudal brainstem and below. *Hoxb8^FlpO^* heterozygous and homozygous mice do not develop any of the sensory or locomotor phenotypes evident in Hoxb8 heterozygous or mutant animals, suggesting normal developmental function of the Hoxb8 gene and protein in *Hoxb8^FlpO^* mice. Compared to the variability of brain recombination in available caudal Cre and Flp lines, *Hoxb8^FlpO^* activity is not present in the brain above the caudal brainstem, independent of mouse genetic background. Lastly, we combine the *Hoxb8^FlpO^* mouse line with dorsal horn developmental lineage Cre mouse lines to express GFP in developmentally determined dorsal horn populations. Using GFP-dependent Cre recombinase viruses and Cre recombinase-dependent inhibitory chemogenetics, we target developmentally defined lineages in the adult. We show how developmental knock-out versus transient adult silencing of the same ROR𝛃 lineage neurons affects adult sensorimotor behavior. In summary, this new mouse line and viral approach provides a blueprint to dissect adult somatosensory circuit function using Cre/Flp genetic tools to target spinal cord interneurons based on genetic lineage.

## Highlights

- A new *Hoxb8^FlpO^* mouse line allows Flp-dependent recombination in the spinal cord, dorsal root ganglia, and caudal viscera.
- We observed no ectopic brain expression across mouse genetic backgrounds with the *Hoxb8^FlpO^* mouse line.
- Combining this new mouse line for intersectional genetics and a viral approach, we provide a novel pipeline to target and manipulate developmentally defined adult spinal circuits.

## Introduction

Lineage-tracing studies have revealed distinct molecularly defined clusters of neural progenitor cells that assemble into circuits responsible for various functions. In the spinal cord, this methodology has led to the identification and genetic targeting of key neural lineages that develop into populations responsible for somatosensory and locomotor behaviors in adult (Jessell, 2000; Lee and Pfaff, 2001; Butler and Bronner, 2015; Lu et al., 2015; Lai et al., 2016). Combining these lineage-defined Cre mouse lines with fluorophores and actuators has allowed key insights into the relationship between cell identity and function. One of the prominent uses of this approach is dissecting somatosensory function, specifically nociception, where manipulations of molecularly defined neuronal populations have various effects on pain processing (Duan et al., 2014; Peirs et al., 2021). However, the anatomical substrates of nociception span across the dorsal root ganglia (DRG) and spinal cord to the brain (Adrienne and Ardem, 2010; Todd, 2010; Mills et al., 2018; Yang and Chang, 2019), suggesting a need for spatial resolution to compare peripheral, spinal, and supraspinal pathways. To address this, the field has utilized intersectional Cre/Flp approaches to selectively target spinal neurons and elucidate their contributions to nociception and sensorimotor integration (Duan et al., 2014; Brian Roome et al., 2020; Barik et al., 2021). A key part of these approaches relies on efficient intersectional targeting, often utilizing molecularly targeting Cre lines with anatomically-restricted developmental Flp lines (Awatramani et al., 2003; Farago et al., 2006; Parkitna et al., 2009; Dymecki et al., 2010). One limitation of intersectional approaches is the cost, availability and specificity of mouse lines targeting an anatomical region of interest. For research looking at spinal contributions to somatosensation and locomotion, mouse lines targeting the spinal cord and DRG are invaluable tools. Fortunately, there are available Flp-recombinase lines that target the DRG (*Advillin^FlpO^*; Choi et al., 2020), caudal neuroectoderm (Cdx2^NSE-FlpO^, Cdx2^FlpO^; Britz et al., 2015; Abraira et al., 2017), or the spinal dorsal horn + dorsal hindbrain (*Lbx1^FlpO^*; Pan et al., 2019), which can be used to target the caudal nervous system. However, expression of these genes can exceed past the spinal cord into the hindbrain, forebrain, and midbrain (Sieber et al., 2007; Coutaud and Pilon, 2013), because of germline recombination depending on the genetic background the line is maintained on, which should be considered for studies conducting brain-sparing manipulations. Therefore, the availability of a brain-sparing mouse line that better restricts expression to the caudal nervous system would greatly benefit researchers looking to elucidate spinal vs. supraspinal contribution to somatosensory and motor function.

The ability to target finer subsets of neuronal populations using intersectional Cre/Flp recombinase systems has allowed for greater insights into cellular function based on molecular and anatomical identity (Britz et al., 2015; Bourane et al., 2015b; Paixão et al., 2019). These intersectional approaches utilize a genetic toolbox of Cre- and Flp-dependent mice that allow for targeted ablation (Duan et al., 2014), chemogenetic manipulation (Ray et al., 2011; Sciolino et al., 2016), and optogenetic access (Hooks et al., 2015; Huang et al., 2018). Although invaluable, one caveat of intersectional approaches is the complex genetic strategies needed to attain intersection-specific expression. For a single intersection, there are often many associated reporters and actuators (Madisen et al., 2015), requiring extensive time and breeding costs to obtain triple or quadruple transgenic animals. Furthermore, intersectional approaches are limited to the availability of dual-recombinase mouse lines, which may not be readily developed for specific usages. Therefore, intersectional strategies are not easily accessible, and can come with a high cost and time barrier. Fortunately, there are a vast number of viral tools available for Cre- and/or Flp-dependent delivery of a multitude of reporters and actuators (Kakava-Georgiadou et al., 2019; Fenno et al., 2020). Taking advantage of the popularity of green fluorescent protein (GFP) mouse lines, Tang et al. (2015) developed a virus which allows Cre recombinase function in the presence of GFP (Cre-DOG). This virus transforms GFP from a reporter into a genetic access point, allowing for the usage of various Cre-dependent viruses. This is particularly useful in developmental populations, where gene expression is transient. One such dynamic environment is the spinal cord, where transient genes are important for the emergence and differentiation of neuronal populations that process somatosensory information (Peirs et al., 2015; Cui et al., 2016; Koch et al., 2017). Therefore, the ability to target populations molecularly defined during development allows for the ability to manipulate the same lineage in the adult, where the gene may no longer be expressed. Furthermore, this strategy can leverage GFP expression in a lineage to allow for viral access, which may not have been previously possible with transient markers.

In order to build on the repertoire of available tools for efficient targeting of adult spinal circuits, we first developed a mouse line to restrict recombinase expression to the spinal cord and caudal tissue, independent of genetic background. We chose to utilize the Flp-FRT recombinase system (Takeuchi et al., 2002), which can be used alongside intersectional Cre-loxP approaches. To achieve spinal targeting, we expressed Flp recombinase under the transcriptional control of mouse homeobox gene *Hoxb8*, expressed from development onwards and with a spatial expression from the caudal brainstem down. Additionally, *Hoxb8^FlpO^* mice are viable and fertile, with no obvious sensory or motor deficits. Therefore, this novel *Hoxb8^FlpO^* mouse line has the advantages of restricted expression to caudal tissue as well as compatibility with existing Cre approaches. To provide an alternative to the costs of intersectional genetics, we developed a combinatorial transgenic and viral strategy that is compatible with constantly updating tools in the field and can be used to target developmental populations in the adult. We utilized a Cre/Flp approach to express GFP in a developmental subset of spinal dorsal horn neurons under the transcriptional control of *Cdx2 or ROR𝛽* (Coutaud and Pilon, 2013; Bourane et al., 2015a) and *Hoxb8*. We next injected a GFP-dependent Cre-DOG virus (Tang et al., 2015) in order to express Cre recombinase in the GFP+ adult neuronal population. With this approach, we enabled expression of Cre recombinase and a Cre-dependent reporter/actuator in developmentally determined adult neurons. Here we were able to use this approach to compare the impact on sensorimotor integration of developmentally silencing ROR𝛃 gene expression versus transiently silencing neurons of the same ROR𝛃 lineage in adult. Therefore, this strategy enables modular manipulation of adult neurons defined by developmental markers for greater insight into the relationship between development and function in sensorimotor processing.

## Materials and methods

### Virus strains

AAV1 pAAV.CAG.LSL.TdTomato (Addgene 100048)

### Plasmids, cell lines, chemicals, and peptides

*Hoxb8^FlpO^* plasmid #1
*Hoxb8^FlpO^* plasmid #2
pCMV^Dsred-FRT-GFP-FRT^ Flp-dependent reporter plasmid
pCAGGS-FLPe Flpe plasmid
pAAV-EF1a-C-CreintG viral plasmid
pAAV-EF1a-N-CretrcintG viral plasmid
Mouse NIH/3 T3 cells

### Experimental mouse lines

R26-FSF-TdTomato (derived from Ai65)
R26-FSF-YFP (derived from Ai57)
R26-LSL-TdTomato (LSL-TdTomato)
FSF-Synaptophysin-GFP (derived from RC::FPSit)
RC::FLTG
*Hoxb8^FlpO^*
*Cdx2^NSE-FlpO^*
*Cdx2^Cre^*

### Key resource table

Mouse anti-NeuN (Millipore MAB377; RRID: AB_2333092)
Rabbit anti-GFP (Invitrogen A11122; RRID: AB_221569)
Rabbit anti-Dsred (Takara 632496)
Chicken anti-GFP (Aves GFP 1020; RRID: AB_10000240)
Mouse anti-CGRP (Sigma C7113)
Rabbit anti-Iba1 (Abcam ab178846)
647 conjugated IB4 (Thermo Scientific/Life Technology I32450)
Alexa Fluor 488 Goat anti-mouse (Life technologies a11001)
Alexa Fluor 488 Goat anti-rabbit (Life Technologies a11034)
Alexa Fluor 488 Goat anti-chicken (Life Technologies a11039)
Alexa Fluor 546 Goat anti-mouse (Life Technologies a11030)
Alexa Fluor 546 Goat anti-rabbit (Life Technologies a11035)

### Software and algorithms

BioRender web software was utilized in the generation of figures. Digigait software was used for plantar paw tracking and gait analysis. Fiji, Zen, and Olympus imaging softwares were used for image processing and histological quantifications. GraphPad Prism software was used for data presentation and statistical analysis.

### Lead contact and materials availability

Further information and requests for resources should be directed to and will be fulfilled by the Lead Contact, Victoria E. Abraira, victoria.abraira@rutgers.edu.

## Experimental model and subject details

Experiments were conducted on mixed background C57Bl/6 (Jackson Laboratory, JAX#000664) and FVB (Charles River Strain#207). Transgenic mouse strains were used and maintained on a mixed genetic background (C57BL/6/FVB). Experimental animals used were of both sexes. All procedures were approved by the Rutgers University Institutional Animal Care and Use Committee (IACUC; protocol #: 201702589). All mice used in experiments were housed in a regular light cycle room (lights on from 08:00 to 20:00) with food and water available *ad libitum*. All cages were provided with nestlets to provide enrichment. All mice were between 1 and 4 months. Animals were co-housed with four mice per cage in a large holding room containing approximately 300 cages of mice.

## Method details

### Generation and validation of the *Hoxb8^FlpO^* mouse line

CRISPR-Cas9 was used to knock-in a T2A-FlpO sequence before the stop codon of Hoxb8. The Hoxb8-FlpO donor plasmid was designed by inserting a T2a sequence (Harris et al., 2014) and FlpO sequence (Raymond and Soriano, 2007) before the stop sequence at the end of *Hoxb8* Exon 2. The donor plasmid contained a 1,987 bp 5′ homology arm sequence, a 1,347 bp T2a-FlpO sequence, and a 1,082 bp 3′ homology arm sequence, flanked by *EcoR1* restriction sites. Next, Cas9 protein (IDT) was complexed with a synthetic sgRNA GCAGAAGGGTGACAAGAAGT (MilliporeSigma) and microinjected with the donor plasmid into pronuclei of C57BL/6 J zygotes. Founders were first genotyped using primers internal to FlpO (FLPOA 5′-TTCAGCGACATCAAGAACGTGGAC-3′AND FLPOB 5′-TCCTGTTCACTCTCTCAGCACG-3′). FlpO positive founders were screened with primers external to the homology arms to determine correct targeting. HOXB8E 5′-GTACCCAGAAGCCAATAGGATGC-3′ and FLPT2AR 5′-TCGAACTGGCTCATTGAGCCTG-3′ were used to screen for 5′ targeting and FLPOA and HOXB8F 5′-TCCTTCAGCCTCAGAATGCAAGG-3′ were used to screen for 3′ targeting.

### Cre-DOG viruses

pAAV-EF1a-C-CreintG (69571) and pAAV-EF1a-N-CretrcintG (69570) viral plasmids were obtained from Addgene. AAV2/1-EF1a-N-CretrcintG and AAV2/1-EF1a-C-CreintG viruses were then produced and concentrated into a single preparation with a titer >5 × 10^12^ GC/mL by Vigene Biosciences.

### Surgical procedures and post-surgical care

#### Spinal cord viral injection

Mice were anesthetized via continuous inhalation of isoflurane (1.5–2.5%) using an isoflurane vaporizer during the surgery. The skin was incised at T12–L3. Paraspinal muscles around the interspace between T12 and 13, T13 and L1 vertebrae were removed and the dura mater and the arachnoid membrane were carefully incised to make a small window to allow the pulled glass pipettes (Wiretrol II, Drummond) to insert directly into the spinal dorsal horn. 150 nL of AAV viruses per injection site were injected bilaterally across two spinal segments (four injection sites total) using a microsyringe pump injector (UMP3, World Precision Instruments).

### Procedures and behavioral testing

Male and female mice on mixed background C57Bl/6 (Jackson Laboratory, JAX#000664) and FVB (Charles River Strain#207) were used for behavioral analyses. Testing was done beginning at 7 weeks of age, and completed by 12 weeks of age. All animals were group housed, with control and mutant animals in the same litters and cages. Littermates from the same genetic crosses were used as controls for each group, to control for variability in mouse strains/backgrounds. Animal numbers per group for behavioral tests are indicated in figures. All behavioral analyses were done by observers blinded to genotype.

#### Allogrooming behavior

Mice were habituated to plastic chambers for 5 min then filmed for 10 min. Behavior such as elliptical, unilateral and bilateral stroke, and body licking was scored.

#### Mechanical sensitivity testing

##### Von frey filaments test

Mice were placed in plastic chambers on a wire mesh grid and stimulated with von Frey filaments using the up-down method (Chaplan et al., 1994) starting with 1 g and ending with 2 g filament as cutoff value.

##### Thermal nociceptive threshold (Hargreaves’s test)

To assess hind paw heat sensitivity, Hargreaves test was conducted using a plantar test device (IITC). Mice were placed individually into Plexiglas chambers on an elevated glass platform and allowed to acclimate for at least 30 min before testing. A mobile radiant heat source of constant intensity was then applied to the glabrous surface of the paw through the glass plate and the latency to paw withdrawal measured. Paw withdrawal latency is reported as the mean of three measurements for both hindpaws with at least a 5 min pause between measurements. A cut-off of 20 s was applied to avoid tissue damage.

##### Digigait automated treadmill

Mice were first acclimated to the Digigait treadmill chamber for 5 min before beginning testing. Following this period, treadmill speed was gradually increased from 0 to 20 cm/s. The plantar placement of mouse limbs were recorded from underneath by a camera. Digigait software was used to track plantar paw placement over time. Appropriate thresholding and manual correction was applied to paw tracking when required. Thresholding was kept consistent between experimental groups. Digigait software utilized paw placement to provide measurements on gait consistency, frequency, duration, and length.

##### Locomotor training

Mice were trained to locomote across a 2 ft. horizontal platform using positive reinforcement for 4 days leading up to data acquisition. Training consisted of minimally stimulated locomotor bouts across the platform until the mouse completed five locomotor bouts without any external stimulation from experimenters. In brief, home cages were placed on one end of the platform and mice placed on the other end. Mice locomoted across the platform at their preferred speed with minimal stimulation and were allowed to climb onto their home cage between walking bouts. Mice were given peanut butter rewards upon completion of their training.

##### Kinematics recordings

Animals were shaved and hindlimb landmarks were labeled with an Oil-Based Paint Marker (Sharpie, Atlanta, GA, United States) on the day of data acquisition. The following five landmarks were labeled: iliac crest, hip joint, knee joint, ankle joint, and metatarsophalangeal (MTP) joint (used as reference for the toe). Locomotor bouts across the horizontal platform were captured with an AOS Technologies PROMON U1000 high speed camera (Tech Imaging Services, Inc., Saugus, MA, United States) at 415 frames per second (FPS). Approximately, 10–12 step cycles (Eisdorfer et al., 2022) were captured in three locomotor bouts. One step cycle is defined by two phases: the stance phase is the time between the initial contact of the hindpaw with the surface of the horizontal platform to the time it lifted off again; and the swing phase consists of the time between when the hindpaw is lifted off of the platform to the time it contacts the platform again. Animals received an intraperitoneal injection (IP) injection of clozapine-N-oxide (CNO) at a dosage of 10 mg/kg 30 min prior to the sessions.

##### Pose estimation

With DeepLabCut (DLC) methods described in Mathis et al. (2018), Nath et al. (2019), and Eisdorfer et al. (2022), we estimated the locations of the iliac crest, hip joint, knee joint, ankle joint, and MTP joint in kinematics recordings. In brief, the hindlimb joints were manually tracked in ~2,700 frames with an image size of 1,778 by 721 px (95% was used to train the ResNet-50-based model; He et al., 2016; Insafutdinov et al., 2016). A *p*-cutoff of 0.6 was used to gage the effectiveness of joint estimation in addition to manual viewing of pose-estimated labeled videos.

#### Kinematics analysis

Using pose estimation files output by custom DLC neural networks, we employed custom code (Python, RStudio) to quantify the following locomotor parameters at the peak of the swing phase: (1) Ankle Joint Height during the swing phase; (2) Knee Joint Angle; and (3) Iliac Crest to MTP Distance: the height of the MTP normalized to the iliac crest. To eliminate pseudoreplication, average values for each parameter were calculated within animals such that group averages were calculated with one value per animal. Data is represented in bar plots with standard error of the mean as error bars.

### Genetic labeling

Cre and Flp activity was evaluated by crossing Cre or Flp male mice to LSL or FSF-reporter females to obtain F1 progeny for histological analysis. Embryos were staged using vaginal plug detection, and the day the plug was identified was considered E0.5.

### Immunohistochemistry

#### Immunohistochemistry of free floating sections

Male and female mice (P30-37) were anesthetized with isoflurane, perfused with 5–10 mL saline-heparin followed by 50 mL 4% paraformaldehyde (PFA) in PBS at room temperature. Vertebral columns were dissected from perfused mice and post-fixed overnight in 4% PFA at 4°C. Spinal cord sections (50 μm thick) were cut on a vibrating blade microtome (Leica VT100S) and processed for immunohistochemistry as previously described (Hughes et al., 2012). In brief, tissue samples were rinsed in 50% ethanol/water solution for 30 min to allow for enhanced antibody penetration. Three washes in a high salt Phosphate Buffer (HS PBS) were conducted each lasting 10 min. The tissue was then incubated in a cocktail of primary antibodies in HS PBS containing 0.3% Triton X-100 (HS PBST) for 48 h at 4°C. Primary antibodies are listed in Key Resources Table. The tissue was washed in HS PBST then incubated in a secondary antibody solution in HS PBST for 24 h at 4°C. Secondary antibodies included an array of species-specific Alexa Fluor 488, 546, and 647 conjugated IgGs (Invitrogen). Isolectin IB4 Conjugated to Alexa 647 dye was used at 1:200 (Invitrogen). The tissue was treated with HS PBST prior to incubation in 4′,6-diamidino-2-phenylindole (DAPI) stain at 1:5,000 dilution. Tissue sections were then mounted on glass slides and coverslipped with Fluoromount Aqueous Mounting Medium (Sigma). The slides were stored at 4°C.

#### Immunohistochemistry of frozen tissue sections

Embryos were post-fixed for 1 h in 4% paraformaldehyde before cryoprotection. Cryosections were taken using a cryostat (Leica CM3050 S) at 12–40 um thickness. Male and female mice (P30-35) were anesthetized with isoflurane, perfused with 5-10 mL saline-heparin followed by 50 mL 4% paraformaldehyde (PFA) in PBS at room temperature. Vertebral columns (including spinal cord and Dorsal Root Ganglions DRGs), heart, liver, kidney, and hindpaw were dissected from perfused mice, post-fixed overnight in 4% PFA at 4°C. DRGs, heart, liver, kidney, and hindpaw were rinsed with 1X PBS, cryoprotected in 30% sucrose, embedded in OCT and frozen at −80°C, then sectioned (12–40 μm thick) using a standard cryostat (Leica CM3050). Spinal cord and brainstem sections were taken at 50 μm, while brain sections were taken at 150 μm. For immunohistochemistry, primary antibodies were diluted in 1X PBS—10% goat serum (Sigma)—3% bovine albumine (Sigma)—0.4% Triton X-100 and incubated overnight at 4°C. Primary antibodies are listed in Key Resources Table. Corresponding species-specific Alexa Fluor 488, 546, and 647 conjugated IgGs (Invitrogen) secondary antibodies were used for secondary detection. Isolectin IB4 Conjugated to Alexa 647 dye was used at 1:200 (Invitrogen).

### Imaging

Images were obtained on either a dissecting scope (Zeiss Stereo Discovery.V8) with mounted camera or a confocal microscope (Zeiss LSM 800 or Olympus Fluoview). Within analyses, imaging parameters and thresholds were kept consistent.

### Quantification and statistical analysis

All data are reported as mean values ± SEM. Behavioral assays were replicated several times (3–10 times depending on the experiments) and averaged per animal. Statistics were then performed over the mean of animals. Statistical analysis was performed in GraphPad Prism (United States) using two-sided paired or unpaired Student’s *t*-tests, one-way ANOVA for neuromorphological evaluations with more than two groups, and one- or two-way repeated-measures ANOVA for functional assessments, when data were distributed normally. *Post hoc* Tukey’s or Bonferroni test was applied when appropriate. The significance level was set as *p* < 0.05. The nonparametric Mann–Whitney or Wilcoxon signed-rank tests were used in comparisons of <5 mice.

### Data and code availability

Data are available upon request from the Lead Contact, Victoria E. Abraira, victoria.abraira@rutgers.edu.

## Results

### Generation of *Hoxb8^FlpO^* mice

**Figure 1:**
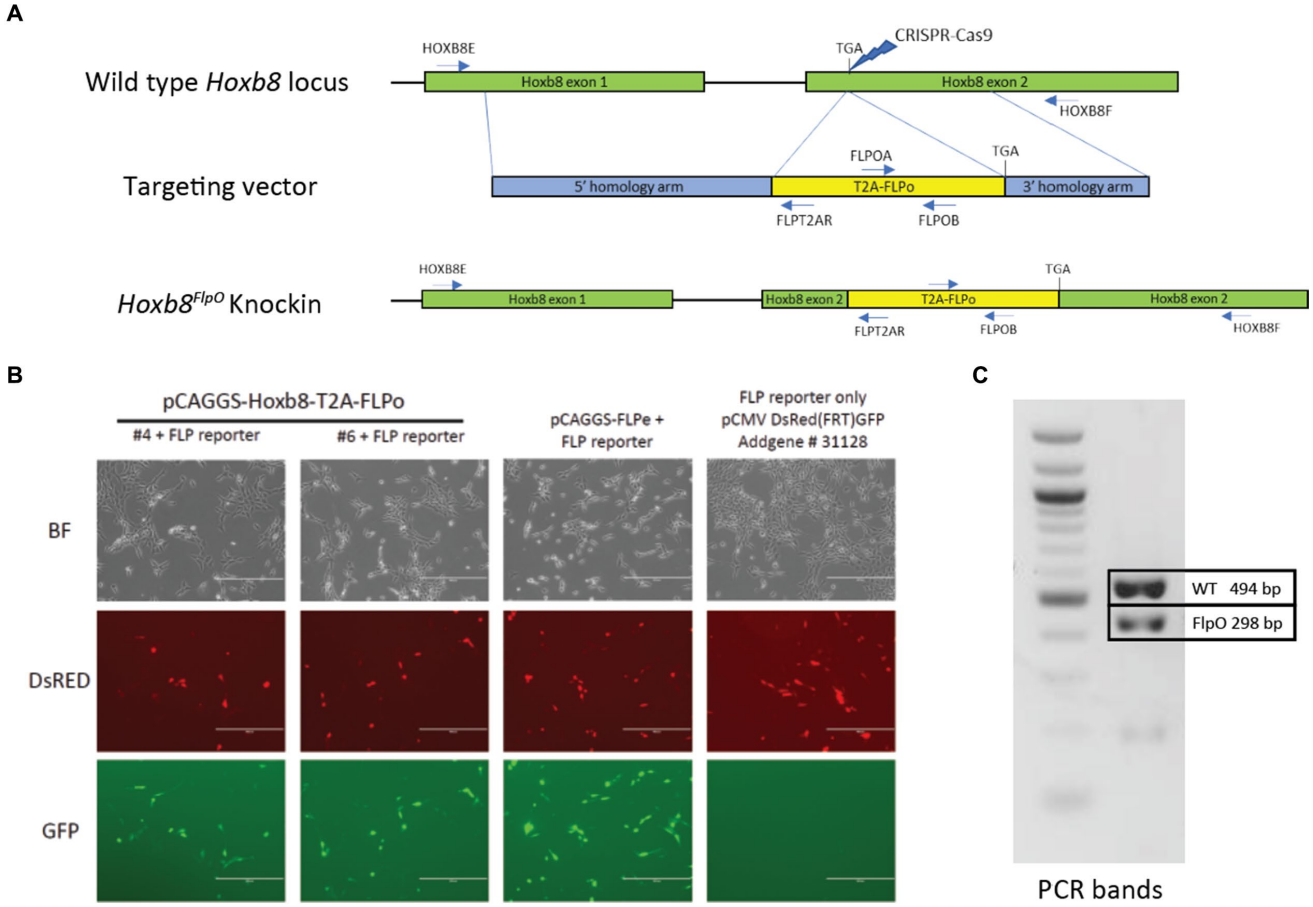
Generation of *Hoxb8^FlpO^* mouse. **(A)** Donor plasmid construct for *Hoxb8^FlpO^* containing a 5′ homology arm (blue), a T2a-FlpO sequence (yellow), and a 3′ homology arm (blue). The construct was targeted for insertion right before the stop codon of *Hoxb8* exon 2 (green). **(B)** Cotransfection of mouse NIH/3 T3 cells by two different *Hoxb8^FlpO^* plasmids along with a Flp-dependent GFP reporter (pCMV^Dsred-FRT-GFP-FRT^). A FlpE plasmid (pCAGGS-FLPe) was used as a positive control. Cells cotransfected with the *Hoxb8^FlpO^* plasmids or FlpE plasmid expressed Flp-mediated GFP recombination, while cells lacking the *Hoxb8^FlpO^* plasmid did not express GFP. Scale bar = 400 μm. **(C)** PCR genotyping results from heterozygous F1 progeny of the founder *Hoxb8^FlpO^* male. One common forward primer, one reverse wild type primer, and one reverse mutant primer were used for the reaction. Results displayed a 494 bp wild type band (top) and a 298 bp mutant band (bottom).

We generated a novel mouse line for flippase-dependent recombination of caudal tissue by targeting the codon-optimized Flp recombinase to the 3′UTR of the mouse homeobox gene *Hoxb8* via CRISPR/Cas9 (Singh et al., 2015). The *Hoxb8* gene was targeted due to its early expression pattern in caudal viscera as well as in spinal and dorsal root ganglia neuron development, and shows a lack of neuronal expression in the brain (Charité et al., 1995; Witschi et al., 2010), making it a strong candidate for targeting spinal circuits. To ensure high levels of Flp recombinase expression and not disrupt endogenous *Hoxb8* gene function, our strategy utilizes a T2A-FlpO gene cassette (Wang et al., 2015) to replace the *Hoxb8* stop codon before the 3UTR (Figure 1A). We evaluated FlpO activity by inserting the T2A-FlpO construct into a high-copy pBluescript plasmid and co-transfected it into mouse NIH/3 T3 cells along with a Flp-dependent GFP reporter pCMV^Dsred-FRT-GFP-FRT^. Cells cotransfected with the *Hoxb8^FlpO^* plasmid expressed Flp-mediated GFP recombination, while cells lacking the *Hoxb8^FlpO^* plasmid did not express GFP (Figure 1B). The donor plasmid contained a 1,987 bp 5′ homology arm sequence, a 1,077 bp 3′ homology sequence, and a T2A sequence in frame with FlpO. Eleven founders had both arms targeted correctly, and one founder was chosen to establish the *Hoxb8^FlpO^* colony. We bred this founder to C57BL/6 J females and used PCR genotyping to identify *Hoxb8^FlpO/+^* F1 progeny (Figure 1C). Progenies of this male will be available at JAX (#037385).

### *Hoxb8^FlpO^* mediated transgene expression is restricted to caudal structures starting embryonic day 9.5.

**Figure 2:**
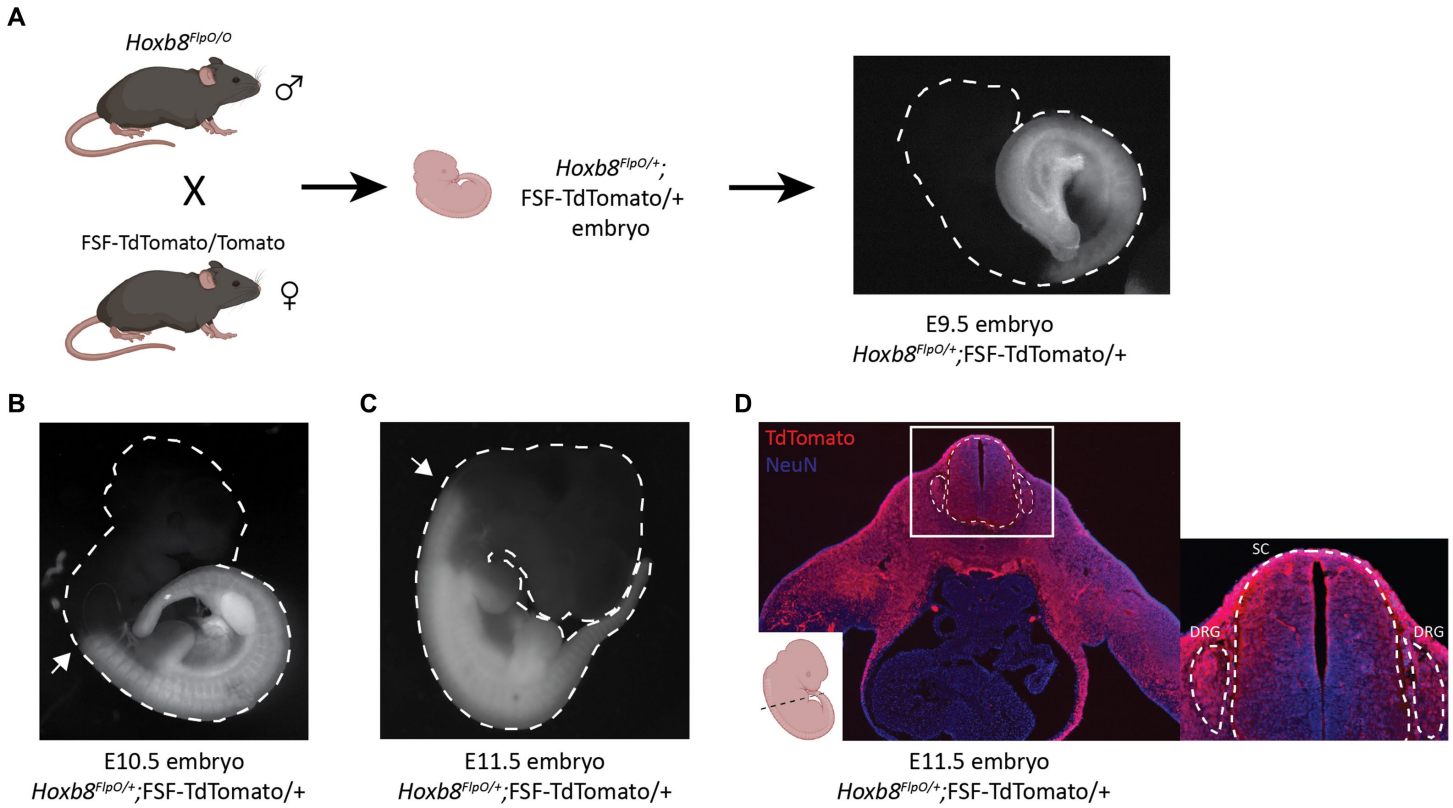
*Hoxb8^FlpO^* mediated transgene expression is restricted to caudal embryonic structures starting at E10.5 of gestation. **(A)** Schematic of breeding scheme for generation of *Hoxb8^FlpO^*;FSF-TdTomato embryos. *Hoxb8^FlpO/O^* males were crossed to FSF-TdTomato/Tomato females, and embryos were dissected from pregnant mothers at E9.5 showing the start of caudal expression pattern of *Hoxb8^FlpO^* during embryonic development. **(B,C)** *Hoxb8^FlpO^*-induced expression of TdTomato in E10.5 **(B)** and E11.5 **(C)** embryos showing caudal expression pattern of *Hoxb8^FlpO^* during embryonic development. At E10.5, the rostral expression boundary of *Hoxb8^FlpO^* ends around somite 10 (arrow), while at E11.5 the rostral boundary extends to around somite 1/2 (arrow). **(D)** Transverse cryosection of E11.5 *Hoxb8^FlpO^*;FSF-TdTomato embryo showing TdTomato fluorescence (red) in viscera, DRG (dashed line), and spinal cord (dashed line). DAPI (blue) was used as a counterstain.

To characterize the developmental pattern of Flp expression, *Hoxb8^FlpO^* mice were crossed to *Rosa26*-FRT-STOP-FRT-TdTomato (FSF-TdTomato) reporter mice derived from the Ai65 mouse line (JAX#021875; Figure 2A). We examined E9.5, E10.5, and E11.5 *Hoxb8^FlpO^*; FSF-TdTomato embryos for expected patterns of *Hoxb8* expression. E10.5 *Hoxb8^FlpO/+^*; FSF-TdTomato/+ embryos exhibited tdTomato fluorescence that was strongly restricted to the caudal neuraxis, gradually disappearing near and rostral to somite 10 (Figure 2B). By E11.5 we see TdTomato fluorescence more rostral in the neuraxis, fading near somite 1 (Figure 2C). TdTomato fluorescence was not observed in *FlpO*^−/−^; FSF-TdTomato/+ littermates. To further assess expression patterns of *Hoxb8^FlpO^* during development, cryostat sections were taken from E11.5 *Hoxb8^FlpO/+^*;FSF-TdTomato mouse embryos. Transverse sections from whole embryos (Figure 2D) reveal strong tdTomato fluorescence in the neural tube and DRG as well as caudal viscera and skin, recapitulating the timeline and location of expected *Hoxb8* gene expression (Charité et al., 1995). These expression patterns are also consistent with those reported for other mouse lines targeting the *Hoxb8* locus (van den Akker et al., 1999; Greer and Capecchi, 2002; Witschi et al., 2010).

### *Hoxb8^FlpO^* mediated transgene expression in the adult nervous system is restricted to neurons of the spinal cord and DRG

**Figure 3:**
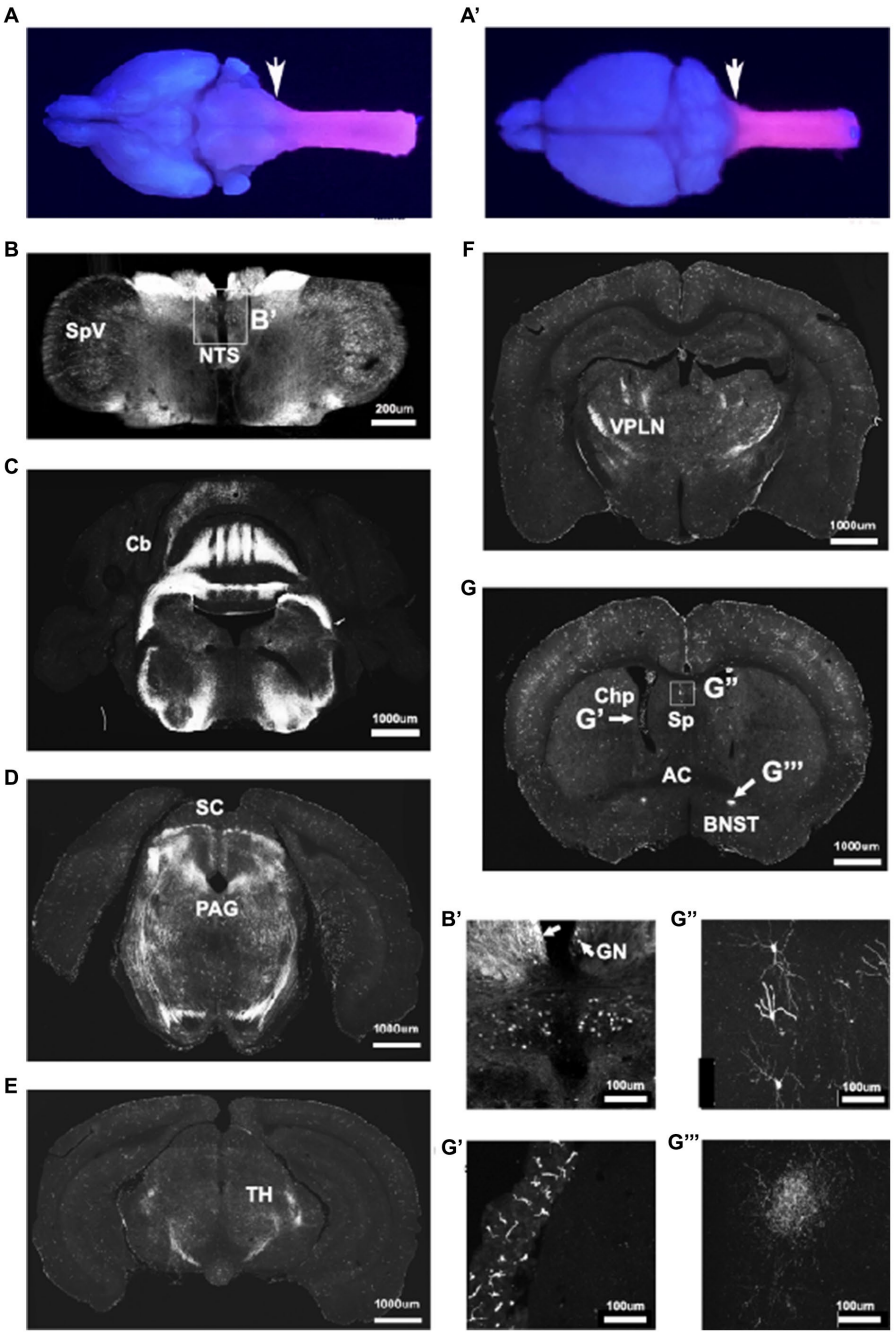
*Hoxb8^FlpO^* expression is restricted to the caudal brainstem and below. Bulbo- and spinofugal axons in the brain of Hoxb8 reporter mice. **(A,A′)** TdTomato reporter in an adult brain and cervical spinal cord visualized under UV illumination. The reporter expression boundary is marked by the arrows. **(B,B′)** Transverse section of the medulla at the level of the obex illustrating positively-labeled neurons in the spinal nucleus of the trigeminal tract (SpV), the nucleus of the solitary tract (NTS), and gracile nucleus (GN, arrows in **B′**). **(C,D)** Transverse sections at the level of the cerebellum (Cb) and superior colliculus (SC, **D**), in which axons are apparent in the periaqueductal gray matter (PAG). **(E,F)** Transverse sections of the caudal **(E)** and rostral **(F)** thalamus showing a dense network of axons in the ventral posterolateral nucleus (VPLN). **(G)** Transverse section at the level of the anterior commissure (AC). Labeled structures include mesenchymal cells in the choroid plexus (Chp, **G′**), scattered neurons in the lateral septal nuclei (Sp, **G′′**) and axons in the fusiform nucleus of the bed nucleus of the stria terminalis (BNST; **G′′′**).

To analyze CNS recombination in adult mice we sectioned tissue from adult P30-37 *Hoxb8^FlpO^*; FSF-TdTomato/+ males and females. UV illumination of whole brains and cervical spinal cords revealed strong fluorescence in the spinal cord and brainstem up to the level of the caudal dorsal column nuclei, reflecting endogenous Hoxb8 expression (Graham et al., 1991; Charité et al., 1995; Ding et al., 2004; Holstege et al., 2008; Figures 3A,A′). Robust input labeling was evident in expected regions of the brainstem, cerebellum, midbrain, and thalamus, major targets of DRG sensory neurons and spinal projection systems (Figures 3B–F). In the hindbrain, the most rostral labeled neurons were in the dorsal column nuclei, the nucleus of the solitary tract, as well as in the spinal nucleus of the trigeminal tract (Figures 3B,B′). The most rostral extent of axon labeling was in the fusiform nucleus of the bed nucleus of the stria terminalis (BNST, Figures 3G,G′′′), which is known to receive a dense projection from the nucleus of the solitary tract (Shi et al., 2021). Axons projecting to the BNST could be followed caudally as far as the thalamus where they were lost among spinothalamic axons. In the telencephalon, there was a scattered population of neurons throughout the rostro-caudal extent of the lateral septum (Figures 3G,G′′). These had fine ventrally-projecting axons that were difficult to follow to their terminals. While many microglia were of a Hoxb8 lineage, they were far less numerous than those previously described in a Hoxb8-IRES-Cre line (De et al., 2018; BNB and MSR, unpublished observations). Hoxb8-lineage mesenchymal cells were also present in the choroid plexus (Figure 3G′).

**Figure 4:**
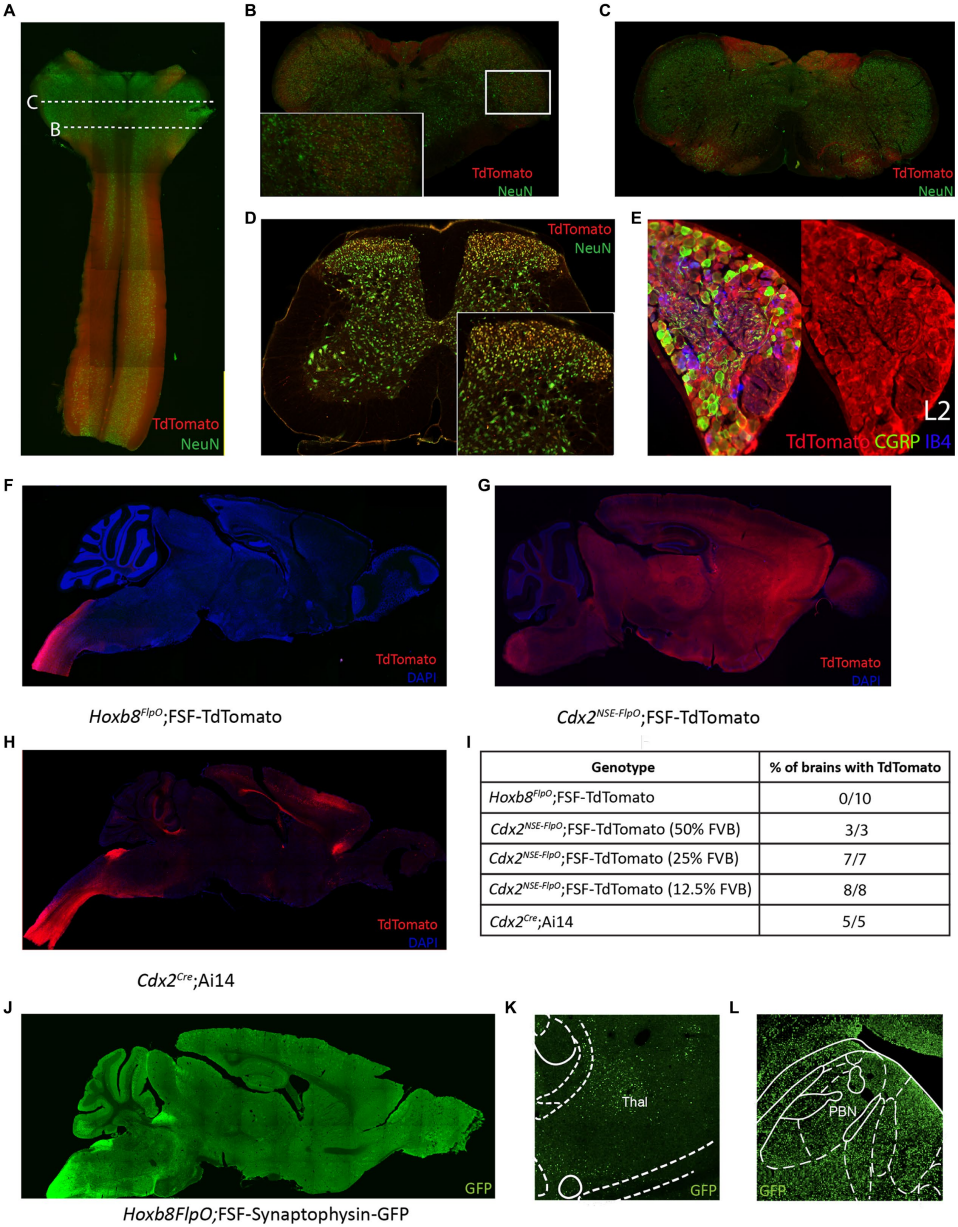
*Hoxb8^FlpO^* mediated transgene expression in the adult is restricted to the spinal cord. **(A)** Sagittal section of cervical spinal cord and brainstem of adult (6 weeks) *Hoxb8^FlpO^*;FSF-TdTomato mouse showing Tdtomato fluorescence (red) largely restricted to the spinal cord. NeuN (green) was used as a counterstain. **(B)** Transverse section of caudal brainstem from an adult *Hoxb8^FlpO^*;FSF-TdTomato mouse showing sparse TdTomato fluorescence (red) in neuronal cell bodies (green) of the spinal trigeminal nucleus. **(C)** Transverse section of rostral brainstem from an adult *Hoxb8^FlpO^*;FSF-TdTomato mouse showing TdTomato fluorescence (red) in neuronal tracts, but not in cell bodies (green). **(D)** Transverse section of lumbar spinal cord from an adult *Hoxb8^FlpO^*;FSF-TdTomato mouse showing TdTomato fluorescence (red) in a large majority of spinal neurons (green). Zoomed in image of spinal neurons is shown in the bottom right. **(E)** Cryosection of an L2 dorsal root ganglia from a *Hoxb8^FlpO^*;FSF-TdTomato mouse showing TdTomato fluorescence (red) in a large majority of DRG neurons, including CGRP+ (green) and IB4+ (blue) neurons. **(F)** Sagittal section of brain and brainstem from an adult *Hoxb8^FlpO^*;FSF-TdTomato mouse showing TdTomato fluorescence (red) in tracts in the brainstem, but absence of TdTomato in neurons of the brain. DAPI (blue) was used as a counterstain. **(G)** Sagittal section of brain and brainstem from an adult *Cdx2^NSE-FlpO^*;FSF-TdTomato mouse on a 25% FVB background showing widespread neuronal TdTomato fluorescence (red) in the brain. DAPI (blue) was used as a counterstain. **(H)** Sagittal section of brain and brainstem from an adult CdxCre;LSL-TdTomato mouse showing TdTomato fluorescence (red) in the brain. DAPI (blue) was used as a counterstain. **(I)** Quantification of the number of brains containing neuronal TdTomato fluorescence in different caudal-targeting mouse lines. Notably, *Hoxb8* tissue had no observable neuronal TdTomato fluorescence, while neuronal TdTomato fluorescence was observed in all *Cdx2^NSE-FlpO^* and *Cdx2^Cre^* samples. **(J)** Image of sagittal brain from adult *Hoxb8FlpO*;FSF-Synaptophysin-GFP mice, showing projections from *Hoxb8+* neurons (green) into the brain. Notably, no GFP+ neurons are observed in the brain. **(K,L)** *Hoxb8^FlpO+^* projections (green) were observed in the thalamus **(K)** and parabrachial nucleus **(L)**, likely reflecting spinothalamic and spinoparabrachial projections, respectively.

In transverse spinal sections, Tdtomato fluorescence was seen throughout the spinal gray matter, present in NeuN+ cells (Figures 4A–D brainstem, D lumbar spinal cord). For quantitative analysis, we performed NeuN immunostaining of adult P30-37 *Hoxb8^FlpO^*; FSF-TdTomato/+ spinal transverse sections. We observed that 89% of NeuN+ cells were TdTomato+ (1,669/1,874, 89.06% ± 0.9%, mean ± SD), this was consistent across the dorsal horn (88.4%), intermediate region (89.4%), and the ventral horn (90.2%). Spinal Iba1+ microglia did not exhibit TdTomato fluorescence (Supplementary Figure S1A), suggesting Flp expression is restricted to neurons and blood vessels in the spinal cord. To assess *Hoxb8^FlpO^* recombination in the DRG, we cryosectioned adult DRGs from *Hoxb8^FlpO^*;FSF-TdTomato mice. TdTomato fluorescence in the lumbar DRG was observed in virtually all DRG neurons—albeit at much lower levels than in spinal neurons—including calcitonin gene-related peptide (CGRP), isolectin B4 (IB4), and NFH-positive neurons, common protein markers used to identify nociceptive and large diameter DRG neurons (Figure 4E; Supplementary Figures S1D,E). Similar patterns of expression were seen in cervical and thoracic DRG (Supplementary Figures S1B,C). Interestingly satellite glial cells seem to also express HoxB8 at low levels.^1^ Next, we assessed *Hoxb8^FlpO^* expression in caudal viscera by taking cryosections of peripheral tissue and organs from adult *Hoxb8^FlpO^*;FSF-TdTomato/+ mice. Cryosections of the metatarsal hindlimb glabrous skin (Supplementary Figure S2A) showed Tomato fluorescence primarily in the subcutis, as well as the muscle, with very sparse labeling in the reticular dermis. Within the kidney (Supplementary Figure S2B), TdTomato fluorescence was present in endothelial and tubular epithelial cells. As expected, no TdTomato fluorescence was observed in the liver (Supplementary Figure S2C) or heart (Supplementary Figure S2D). This expression pattern recapitulates both *in situ* hybridization and immunohistochemistry results against *Hoxb8* (Deschamps and Wijgerde, 1993) from previous studies (van den Akker et al., 1999; Witschi et al., 2010).

To compare *Hoxb8^FlpO^* expression to existing caudal-targeting recombinase mouse lines, we took coronal brain sections from *Hoxb8^FlpO^*;FSF-TdTomato (Figure 4F), *Cdx2^NSE-FlpO^*;FSF-TdTomato (Figure 4G), and *Cdx2^Cre^*;R26-LSL-TdTomato (Ai14, JAX#007914; Figure 4H) mice (Hinoi et al., 2007; Abraira et al., 2017). The *Cdx2^Cre^* mouse line was generated by using a human caudal type homeobox 2 promoter/enhancer sequence driving expression of a nuclear-localized Cre recombinase (Coutaud and Pilon, 2013). The *Cdx2^NSE-FlpO^* mouse line was generated by using a fragment of intron 1 of the mouse *Cdx2* gene along with a neural specific enhancer sequence driving Flp recombinase expression (Abraira et al., 2017). The *Cdx2^NSE-FlpO^* line is recommended to be maintained on a 50% FVB background for proper expression, reflecting the fact that the regulatory elements used to generate this line were cloned from FVB genomic DNA (Coutaud and Pilon, 2013). However, it can be difficult to maintain complex double or triple transgenic intersections on a specific background. Therefore, to see how well the *Cdx2^NSE-FlpO^* line functioned on a lesser FVB background, we assessed the efficiency of *Cdx2^NSE-FlpO^* on a 50, 25, or 12.5% FVB background. We took coronal brain sections from *Hoxb8^FlpO^*;FSF-TdTomato, *Cdx2^NSE-FlpO^*;FSF-TdTomato and *Cdx2^Cre^*;LSL-TdTomato (Figure 4I) mice to compare TdTomato fluorescence in the brain. Here, neuronal TdTomato fluorescence was observed in 0/10 *Hoxb8^FlpO^*;FSF-TdTomato brains (Supplementary Figure S2H), with sparse labeling observed within glial cells primarily in the cortex (Supplementary Figure S2E), consistent with established *Hoxb8* expression patterns (Nagarajan et al., 2017). Comparatively, 3/3, 7/7, and 8/8 *Cdx2^NSE-FlpO^*;FSF-TdTomato brains exhibited broad and non-specific neuronal TdTomato fluorescence when mice were bred on a 50, 25, and 12.5% FVB background, respectively, (Supplementary Figures S2I,J). Furthermore, 5/5 *Cdx2^Cre^*;LSL-TdTomato brains exhibited neuronal TdTomato fluorescence in the brain, largely in the cortex (Supplementary Figure S2F) and sparsely in the parabrachial nucleus (PBN; Supplementary Figure S2G).

One main facet of studying pain and itch is the ability to target projection neurons, so we assessed if the *Hoxb8^FlpO^* line can be used to study spinal projections to the brain. We utilized *Hoxb8^FlpO^*;FSF-Synaptophysin-GFP/+ (derived from RC::FPSit mice, JAX# 030206) to label synaptic projections from *Hoxb8* tissue (Figure 4J). We observed synaptic inputs throughout the brainstem, hindbrain, and cerebellum. Notably, we observed GFP+ puncta in the thalamus (Figure 4K) and PBN (Figure 4L), representing spinothalamic and spinoparabrachial projections, respectively. Collectively, this suggests that *Hoxb8^FlpO^* can be utilized to efficiently target the caudal nervous system without having obvious strong background effects.

### Normal spinal cord development and somatosensory function in *Hoxb8^FlpO^* heterozygous and homozygous animals

**Figure 5:**
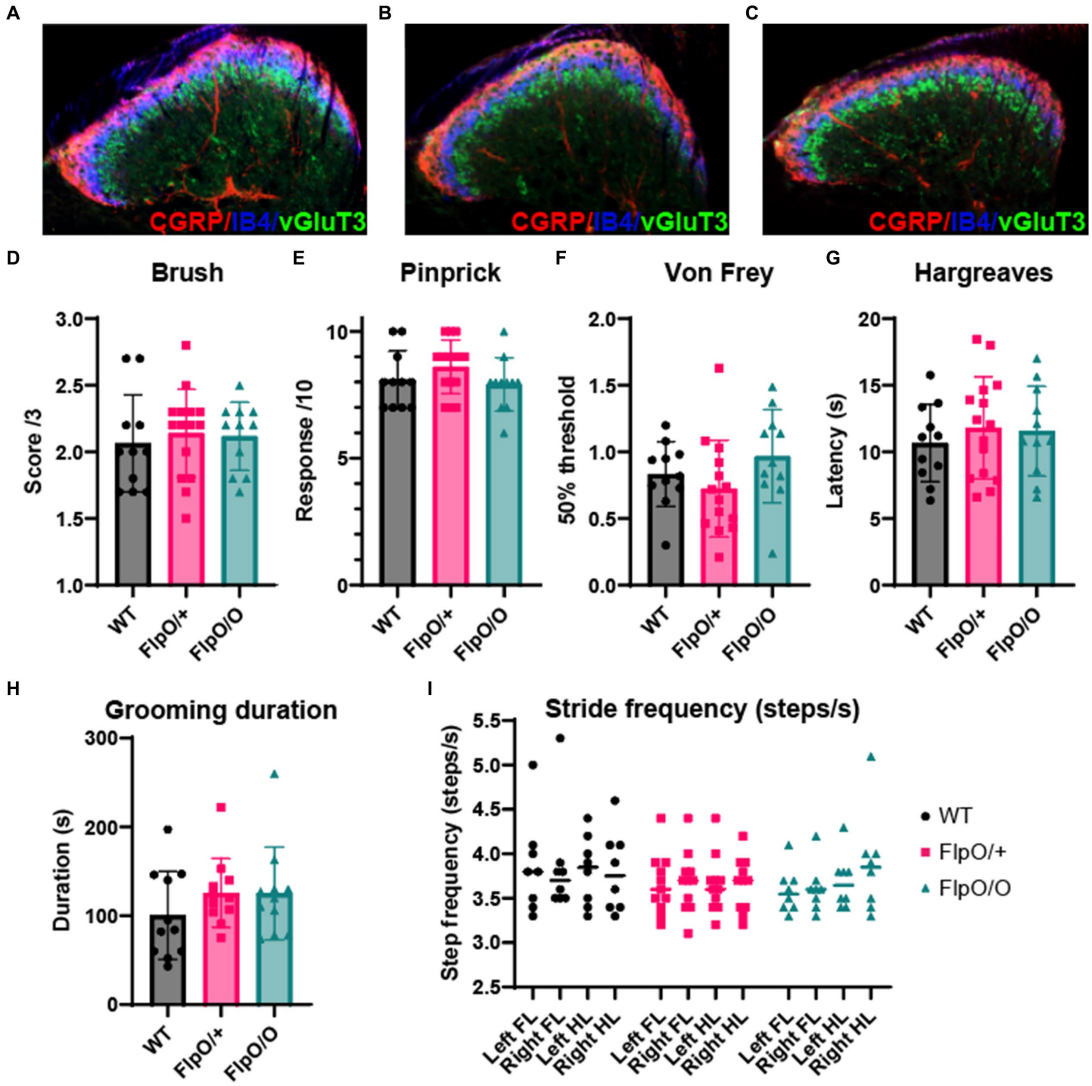
*Hoxb8^FlpO^* mice exhibit normal spinal cord development and somatosensory function. **(A–C)** Immunostaining for different laminae markers in transverse lumbar sections from *Hoxb8^FlpO/O^* **(A)**, *Hoxb8*^*FlpO/*+^ **(B)**, and wild type (WT; **C**) mice reveals no obvious differences in laminae formation defined by immunostaining for lamina markers. Staining was conducted for CGRP (lamina I-red), IB4 (lamina IIo-blue), and VGLUT3 (lamina IIi-green). *n* = 3 mice per group. **(D–G)** Sensory assays used to probe mechanical and heat sensitivity in *Hoxb8*^*FlpO/*+^ mice. *Hoxb8^FlpO/O^* and *Hoxb8*^*FlpO/*+^ mice have no differences in withdrawal response to dynamic brush **(D)**, noxious pinprick **(E)**, von Frey **(F)**, or radiant heat **(G)** compared to wild type littermate controls, suggesting normal mechanical and heat sensory processing. Data are represented as mean ± SEM (*n* = 11–15 mice per group), and significance was assessed using a one-way ANOVA with Tukey’s multiple comparisons. **(H)** *Hoxb8*^*FlpO/*+^ and *Hoxb8*^*FlpO/*+^ mice have no differences in time spent self-grooming compared to wild type littermates, suggesting normal grooming behavior. Data are represented as mean ± SEM (*n* = 10–11 mice per group), and significance was assessed using a one-way ANOVA with Tukey’s multiple comparisons. **(I)** *Hoxb8^FlpO/O^* and *Hoxb8*^*FlpO/*+^ mice have no difference in stride frequency compared to wild type littermates. Data are represented as mean + individual data points (*n* = 9–15 mice per group), and significance was assessed using a one-way ANOVA with Tukey’s multiple comparisons.

Deletion of the *Hoxb8* allele during development results in abnormal dorsal horn laminae formation and sensory defects (Holstege et al., 2008), abnormal limb movements (van den Akker et al., 1999), and excessive grooming behavior (Greer and Capecchi, 2002; Chen et al., 2010). To ensure that insertion of the T2A-FlpO sequence into the 3′UTR of the Hoxb8 gene did not affect endogenous *Hoxb8* activity, and by association normal spinal cord development and function, we first assessed dorsal horn lamination in heterozygous and homozygous *Hoxb8^FlpO^* mice compared to wild type littermates. We took transverse sections from lumbar spinal cords of wild type (Figure 5A), *Hoxb8*^*FlpO*/+^ (Figure 5B), and *Hoxb8^FlpO/O^* (Figure 5C) male and female mice and used immunohistochemistry to stain for markers defining lamina I (CGRP), lamina IIo (IB4), and lamina IIi (vGlut3). No observable difference was seen in dorsal horn lamination between *Hoxb8*^*FlpO*/+^ and *Hoxb8^FlpO/O^* lumbar sections compared to the wild-type sections. Next, given the ubiquitous expression pattern of Hoxb8 in the spinal cord and dorsal root ganglia, we assessed if the T2A-FlpO insertion had any effect on sensory or motor function in *Hoxb8*^*FlpO*/+^ and *Hoxb8^FlpO/O^* male and female mice. Both heterozygous and homozygous mice had no difference in sensitivity to dynamic brush (Figure 5D), pinprick (Figure 5E), von Frey (Figure 5F), or Hargreaves (Figure 5G) stimulation of the plantar hindlimb compared to wild type littermates, suggesting normal tactile and heat sensitivity. Given that *Hoxb8* manipulation disrupts proper grooming (Greer and Capecchi, 2002), we next isolated and recorded individual mice to determine if T2A-FlpO insertion affected self-grooming behavior. *Hoxb8*^*FlpO*/+^ and *Hoxb8^FlpO/O^* mice spent a comparable amount of time grooming themselves compared to wild type littermates (Figure 5H). Next, due to evidence that Hoxb8 mutations can result in aberrant limb reflexes (van den Akker et al., 1999), we tested limb reflexes and found no evidence of hindlimb clasping or other aberrant reflexes in *Hoxb8*^*FlpO*/+^ or *Hoxb8^FlpO/O^* mice. Lastly, *Hox* genes have been implicated in proper motor neuron development and locomotor function (Catela et al., 2022). To assess motor function, we used the Digigait automated treadmill (Dorman et al., 2014) to analyze locomotor gait parameters in *Hoxb8*^*FlpO*/+^ and *Hoxb8^FlpO/O^* mice. We found no significant difference across groups in stride frequency (Figure 5I), stride length (Supplementary Figure S3A), or stride duration (Supplementary Figure S3B), suggesting that T2A-FlpO insertion into the Hoxb8 locus does not affect Hoxb8 gene function in the development of normal spinal cord-mediated locomotion.

### Cre-DOG virus to manipulate adult spinal neuronal populations defined by developmentally expressed transcription factors

Current intersectional genetic approaches utilize a breeding strategy involving genetic intersections crossed to a multitude of dual recombinase-dependent reporters and effectors (Ray et al., 2011; Bang et al., 2012; Madisen et al., 2015; Plummer et al., 2015; Niederkofler et al., 2016). This paradigm can lead to high maintenance and breeding costs, often requiring the long-term maintenance of breeders for multiple intersections and dual-recombinase mice. For a given intersection, one needs access to Cre-dependent reporters (Madisen et al., 2009), Flp-dependent reporters (Sousa et al., 2009), dual Cre/Flp-dependent reporters (Yamamoto et al., 2009; Madisen et al., 2015; Daigle et al., 2018), and a host of different effector mouse lines for ablation (Buch et al., 2005), activation (Zhu et al., 2016), silencing (Zhu et al., 2016), or optogenetics (Madisen et al., 2012). Therefore, this strategy requires the maintenance of a large animal colony for an extended period of time. The ability to visualize and manipulate molecularly defined neurons in a modular fashion would drastically cut down on breeding costs and allow for more simple breeding strategies. Additionally, this would be useful to study developmental populations, where gene expression can be transient during a developmental time window and absent in adult. Due to this transient expression, manipulating early developmental genes to understand their functional contributions are confounded by potential developmental abnormalities or compensatory mechanisms (Matthaei, 2007; Knockout Mice Fact Sheet, 2022). Therefore, a strategy to manipulate molecularly determined neuronal populations in the adult would enable the comparison of developmental phenotypes to those in the mature animal.

**Figure 6:**
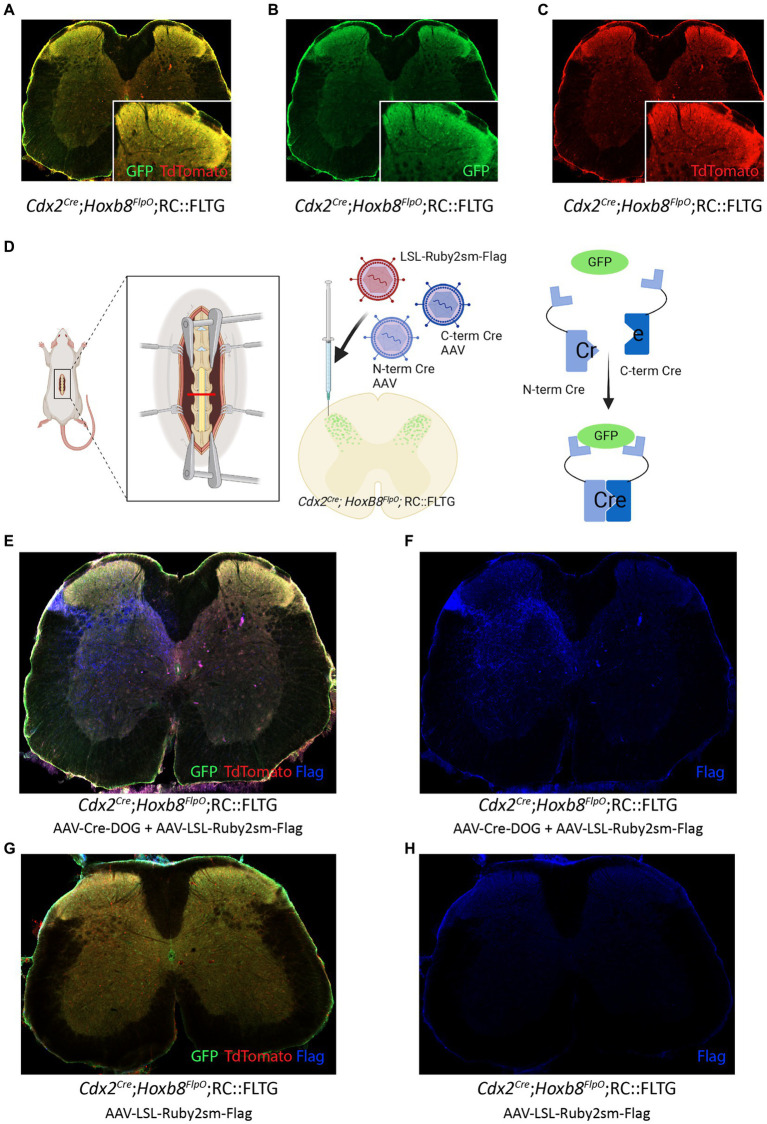
Cre-DOG virus to label spinal cord lineages defined by Cdx2 and Hoxb8. **(A,B,C)** Expression of GFP and Tomato in a transverse lumbar section of a *Cdx2^Cre^*;*Hoxb8^FlpO^*;RC::FLTG mouse. **(D)** Schematic of Cre-DOG virus strategy in the *Cdx2^Cre^*;*Hoxb8^FlpO^*;RC::FLTG mouse. The Cre-DOG virus consists of N- and C-terminal Cre fragments, which combine and become active in the presence of GFP. In *Cdx2^Cre^*;*Hoxb8^FlpO^*; RC::FLTG mice, endogenous *Cdx2^Cre^* expression is absent in the adult. Therefore, injection of the Cre-DOG virus drives expression of Cre in GFP+ cells, in the absence of endogenous *Cdx2^Cre^* expression. **(E,F,G,H)** Transverse lumbar image from *Cdx2^Cre^*;*Hoxb8^FlpO^*;RC::FLTG mice co-injected with AAV-Cre-DOG and AAV-LSL-Ruby2sm-Flag **(E,F)** or AAV-LSL-Ruby2sm-Flag only **(G,H)**. Cre-DOG-mediated Cre expression becomes active in GFP+ cells, which drives expression of the viral LSL-Ruby2sm-Flag, labelled in blue in E,F but not in G,H where there is no Cre expression. **(G,H)** Transverse image from a *Cdx2^Cre^*;*Hoxb8^FlpO^*;RC::FLTG mouse injected with AAV-LSL-Ruby2sm-Flag. Cre-dependent Flag expression is not observed in this tissue due to the lack of cdx2-driven Cre expression in the adult mouse.

One gene important for normal spinal development and rostrocaudal patterning is *Cdx2* (van den Akker et al., 2002; Mazzoni et al., 2013), which is transiently expressed in both the dorsal and the ventral horn in early embryo development at E8 and absent in adult. Studies have identified that *Cdx2* is necessary for proper acquisition of caudal nervous system neural identity (Metzis et al., 2018) by inducing Hox gene expression in neural progenitors, including motor neurons. Furthermore, *Cdx2* has been utilized in intersectional approaches to target neurons implicated in mechanical hypersensitivity (Orefice et al., 2016, 2019; Abraira et al., 2017; Chirila et al., 2022; Meltzer et al., 2023), pain (Brian Roome et al., 2020; Choi et al., 2020), itch (Escalante and Klein, 2020) and locomotion (Bourane et al., 2015b). To further study the pattern of expression of *Cdx2* and its overlap with *Hoxb8*-lineage neurons, we developed a method to target *Cdx2*-lineage neurons in the adult. We crossed *Cdx2^Cre^*;*Hoxb8^FlpO^* mice (Coutaud and Pilon, 2013) to RC::FLTG mice (JAX#026932), where Flp-recombinase induces TdTomato fluorescence, and additional Cre recombinase expression induces GFP fluorescence. Within the *Cdx2^Cre^*;*Hoxb8^FlpO^*;RC::FLTG intersection, neurons that have expressed both *Hoxb8* and *Cdx2* in the spinal cord will be labeled with GFP (Figures 6A,B). TdTomato fluorescence was also observed in this intersection due to Flp activity in *Hoxb8*-lineage neurons (Figure 6C). This overlap between GFP and TdTomato fluorescence suggests *Hoxb8^FlpO^* targets the same broad population of spinal neurons as the *Cdx2^Cre^* mouse line. To show we can combine intersectional viral and genetic strategies to target this developmentally-determined population in adult, we used a viral tool AAV-Cre-DOG (Tang et al., 2015) consisting of N- and C-terminal fragments of Cre recombinase which combine and become functional solely in the presence of GFP in a cell (Figure 6D). The AAV-Cre-DOG virus was co-injected with a Cre-dependent Spaghetti Monster virus (AAV-CAG-LSL-Ruby2sm-Flag, Addgene 98928-AAV1) into the lumbar spinal cord. Spaghetti monster constructs are not fluorescent but we were able to detect FLAG expression in adult Cdx2-lineage neurons (Figures 6E,F). Due to the lack of *Cdx2* expression in the adult spinal cord (Xue et al., 2010), the observed FLAG immunoreactivity is expected to arise from the viral Cre-DOG approach. To control for unexpected *Cdx2^Cre^* expression in the adult, we injected adult (P56-98) *Cdx2^Cre^*;*Hoxb8^FlpO^* mice with the same Cre-dependent AAV-CAG-LSL-Ruby2sm-Flag (Figures 6G,H). We observed no FLAG immunoreactivity in this tissue, suggesting that FLAG expression is indeed induced by the AAV-Cre-DOG and not the *Cdx2^Cre^*. Using this approach, we were able to target Cre expression to adult *Cdx2*-lineage neurons in the spinal cord, which can then be further probed using Cre-dependent viral tools.

### Intersectional viral-genetic approaches to dissect developmentally determined adult somatosensory circuit function

After successfully using our viral-genetic approach to label a developmentally-determined neuronal population in the adult, we applied this approach to further probe the adult function of a developmentally-determined neuronal circuit important for sensorimotor integration (Wiltschko et al., 2015; Koch et al., 2017). Loss of gene function during development can have adverse effects on sensory and locomotor function (Gross et al., 2002; Watson et al., 2015; Koch et al., 2017; Masgutova et al., 2019). One example is the gait deficits observed in spinal knockouts of the *Ror𝛽* gene, which is hypothesized to regulate sensory feedback during locomotion (Koch et al., 2017). *Ror𝛽* encodes the retinoic acid-related orphan nuclear hormone receptor *𝛽* in a mixed population of deep (medial laminae V-VI) and more superficial (laminae IIi-IV) dorsal horn neurons (Koch et al., 2017). This mixed superficial and deep dorsal horn population encompasses a large number of neurons during development (P0 onwards) and progressively gets restricted to a smaller number of neurons in adult (Koch et al., 2017). Global knockout of the gene and spinal ablation of developmentally-determined *Ror𝛽* neurons results in a characteristic “duck gait” phenotype (Wiltschko et al., 2015; Koch et al., 2017), where aberrant proprioceptive feedback leads to impaired locomotion. However, both developmental manipulations and neuronal ablation of the *Ror𝛽* developmental lineage have caveats such as abnormal developmental effects and compensatory mechanisms during neuronal death. Moreover, *Hoxb8* is expressed in spinal neurons but absent from spinal microglia and astrocytes, while *Ror𝛽* is expressed in astrocytes (RNAsequencing data, Linnarsson lab, http://mousebrain.org/adolescent/genesearch.html). Thus the *Ror𝛽^Cre^*;*Hoxb8^FlpO^* intersection strategy allows us to target *Ror𝛽* spinal neurons. Therefore, we wanted to see how reversibly silencing the developmental versus the adult *Ror𝛽+* neuronal populations would affect locomotion in relation to the “duck gait” phenotype.

**Figure 7:**
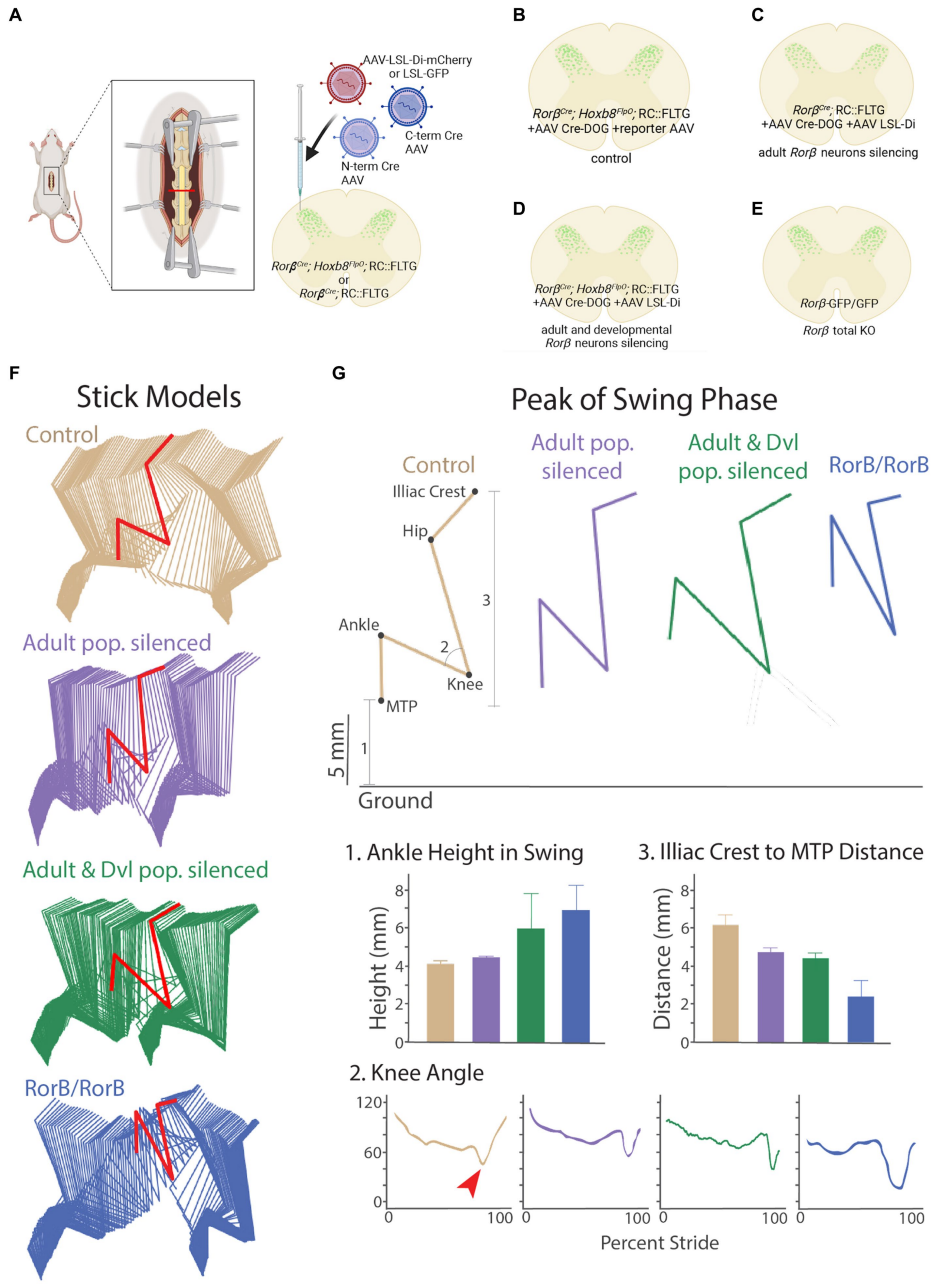
CRE-DOG and inhibitory chemogenetics viruses to assess developmentally determined *ROR𝛽* lineage neurons to sensorimotor integration in adult. **(A)**. Schematic of Cre-DOG virus strategy in the *Ror𝛽^Cre^*;*Hoxb8^FlpO^*;RC::FLTG mouse. The Cre-DOG virus consists of N- and C-terminal Cre fragments, which combine and become active in the presence of GFP, and a Cre-dependent LSL-Di-mCherry virus. **(B–E)** Schematic of the *Ror𝛽* populations silenced in each experimental group. **(B)** Control animals with dorsal horn injection of AAV-Cre-DOG and control reporter virus LSL-eGFP **(C)**. The *Ror𝛽^Cre^* gene is expressed during development more broadly than in adult mice in the superficial and deeper dorsal horn. As a consequence, *Ror𝛽^Cre^*;RC::FLTG + AAV Cre-DOG + AAV LSL-Di-mCherry silences the adult *Ror𝛽* populations (ad-*Ror𝛽*-Di). **(D)** *Ror𝛽^Cre^*;*Hoxb8^FlpO^*;RC::FLTG + AAV Cre-DOG + AAV LSL-Di-mCherry silences the adult and developmental lineage *Ror𝛽* populations (ad+dev-*Ror𝛽*-Di). **(E)** *Ror𝛽^GFP/GFP^* is a developmental total Knock-Out of the *Ror𝛽^Cre^* gene. **(F)** Stick model representations (two step cycles) of control (tan), ad-*Ror𝛽*-Di (purple), ad+dev-*Ror𝛽*-Di (green), and *Ror𝛽^GFP/GFP^* (blue) mutant groups. Locomotor parameters were quantified in the peak of the swing phase (red). (**G**; Top) Example stick model representation of the peak of the swing phase. (Bottom) The following locomotor parameters were quantified: **(1)** Ankle Joint Height during the swing phase; **(2)** Knee Joint Angle (red arrow points to the peak of swing phase); and **(3)** Iliac Crest to MTP Distance. Control mice exhibit the smallest Ankle Height, largest Knee Angle, and largest Iliac Crest to MTP Distance. *Ror𝛽^GFP/GFP^* mice exhibit the highest Ankle Height, smallest Knee Angle, and smallest Iliac Crest to MTP Distance due to their characteristic “duck gait” phenotype. Following administration of DREADDs-agonist CNO, ad-*Ror𝛽*-Di and ad+dev-*Ror𝛽*-Di mice display intermediate phenotypes, with ad+dev-*Ror𝛽*-Di mice exhibiting the more severe phenotype of the two.

To address this, we utilized a *Ror𝛽^Cre^*;*Hoxb8^FlpO^*;RC::FLTG intersectional genetic strategy combined with AAV-Cre-DOG to manipulate the activity of all *Ror𝛽*-lineage neurons and compare it to the manipulation of the *Ror𝛽*-lineage neurons that retain *Ror𝛽* expression into adulthood. We first co-injected AAV-Cre-DOG and a Cre-dependent inhibitory DREADD virus AAV1-hSyn-DIO-hM4D(Gi)-mCherry (AAV-LSL-Di) into the lumbar spinal cord of adult *Ror𝛽^Cre^*;*Hoxb8^FlpO^*;RC::FLTG and *Ror𝛽^Cre^*;RC::FLTG mice (Figure 7A). We used a Cre-dependent reporter virus as viral control (Figure 7B; Supplementary Figure S4A), as well as animals negative for Cre and FlpO, and hence lacking GFP expression, as genetic control. This led to expression of an inhibitory DREADD receptor in the developmentally determined *Ror𝛽*-lineage spinal neurons, which includes the adult neuron population that retain the expression of *Ror𝛽* (referred to as ad + dev-*Ror𝛽*-Di mice, Figure 7D; Supplementary Figure S4C), or in the adult *Ror𝛽+* population only (referred to as ad-*Ror𝛽*-Di mice; Figure 7C; Supplementary Figure S4B), respectively. With the application of DREADDs agonist clozapine-N-oxide (CNO; Roth, 2016), we identified gait deficits of ad-*Ror𝛽*-Di and ad + dev-*Ror𝛽*-Di mice and compared their locomotor behaviors to control animals and *Ror𝛽* mutant mice (*Ror𝛽* ^GFP/GFP^, “duck gait,” Figure 7E; Supplementary Videos S1–S4). Natural walk (Fiander et al., 2017) was captured across a flat horizontal surface using a high-speed camera and by labeling the following hindlimb landmarks: the iliac crest, hip joint, knee joint, ankle joint, and metatarsophalangeal (MTP) joint (used to reference the toe; Figure 7F). It has been previously demonstrated that the “duck gait” phenotype of *Ror𝛽* mutant mice (*Ror𝛽* ^GFP/GFP^) can be quantified during the swing phase of a step cycle (Koch et al., 2017). More specifically, we observed that gait deficits of ad-*Ror𝛽*-Di, ad + dev-*Ror𝛽*-Di, and *Ror𝛽* mutant mice are pronounced during the peak of the swing phase (Figures 7F,G). We quantified three distinct locomotor parameters to describe this phenomenon (Figure 7G): (1) Ankle Joint Height during the swing phase; (2) Knee Joint Angle; and (3) Iliac Crest to MTP Distance: the height of the MTP normalized to the iliac crest. Using these parameters, we observed that control animals exhibit the smallest Ankle height, largest Knee Angle, and largest Iliac Crest to MTP Distance. As a direct result of their “duck gait” phenotype, we observed *Ror𝛽* mutant mice exhibit the highest Ankle height, smallest Knee Angle, and smallest Iliac Crest to MTP Distance. In the presence of CNO, we observed intermediate phenotypes for ad-*Ror𝛽*-Di and ad + dev-*Ror𝛽*-Di mice. As expected, silencing both adult and developmentally determined *Ror𝛽* -lineage neurons (ad + dev-*Ror𝛽*-Di mice) resulted in a more drastic gait deficit than silencing the adult *Ror𝛽* + neurons alone (Figures 7F,G). Our results demonstrate that our viral-genetic approach to label a developmentally-determined neuronal population in the adult can have quantifiable implications on functional behavior. Therefore, this approach can be utilized to investigate the function of developmentally determined neurons and compare it to the role of populations arising at later developmental time points, without altering gene function at key developmental stages.

## Discussion

A key advantage of mouse genetic strategies is the ability to restrict reporter or effector gene expression to a molecularly defined subset of neurons. This genetic gateway can be utilized to study neurons based on their identity or geography, allowing for targeted manipulations with high specificity. For example, the study of nociception has benefitted from the ability to conduct global or conditional manipulations using the Cre/loxP system (Gingras et al., 2014; Chen et al., 2018; Boinon et al., 2022). More recent studies have utilized intersectional genetic strategies, which allow for even finer manipulations of neurons of interest (Duan et al., 2014; Barik et al., 2021). Although powerful, one caveat of this approach is the availability of specific mouse lines of interest, such as restrictions that selectively target the DRG, spinal cord, or brain. Although there are lines that target sensory ganglia (Zhou et al., 2002; Lau et al., 2011) or subsets of primary afferents (Stirling et al., 2005; Vrontou et al., 2013; Abraira et al., 2017), there are relatively fewer tools available to specifically target the spinal cord and DRG while sparing the brain. In order to determine spinal versus supraspinal contributions to pain, there is a constant need for the evolution of genetic tools to meet the demand of more specific manipulations. To generate a tool for better brain-sparing intersections, we developed a *Hoxb8^FlpO^* mouse line where *Flp* recombinase expression is largely restricted to spinal and DRG neurons, as well as caudal viscera. We show that *Hoxb8^FlpO^* mice exhibit Flp recombinase expression in the intended, brain-sparing pattern and are viable and fertile. Importantly, these mice can be utilized in combination with existing Cre lines for intersectional approaches to generate finer, brain-sparing intersections to study the contributions of spinal and DRG neurons to mediating nociception.

In order to develop a strategy to utilize brain-sparing recombinases in the study of pain and itch, we compared *Hoxb8^FlpO^* expression to other caudal-targeting mouse lines. As expected in *Hoxb8^FlpO^*;FSF-TdTomato tissue, we saw no neuronal TdTomato fluorescence in the brain, reflecting the endogenous expression pattern of *Hoxb8* (Deschamps and Wijgerde, 1993). However, we observed ectopic brain expression in both *Cdx2^NSE-FlpO^*;FSF-TdTomato and *Cdx2^Cre^*;LSL-TdTomato tissue, suggesting careful consideration in their usage as brain-sparing lines. The *Cdx2^NSE-FlpO^* line was developed by taking a 852 bp neural specific enhancer sequence of intron 1 in the mouse *Cdx2* gene, and cloning it upstream of a FlpO cassette. One likely source of ectopic brain expression for this line is related to genetic background. Since the regulatory sequences used during the cloning were from FVB genomic DNA, this line is recommended to be maintained on a FVB background. The difficulty of maintaining the recommended minimum 50% FVB background during complex intersectional genetic strategies is a main caveat of the *Cdx2^NSE-FlpO^* line. Here, we show the efficiency of the *Cdx2^NSE-FlpO^* when maintained on a 50%, 25% FVB, or 12.5% FVB background (Figures 4G,I), where we observed ectopic brain expression in both cases. We observed similar levels of ectopic brain expression in *Cdx2^Cre^*;LSL-TdTomato mice (Figures 4H,I). However, ectopic brain expression was different across these two lines: for the *Cdx2^Cre^* we largely saw brain expression in the cortex (Figure 4H; Supplementary Figure S1I), while in *Cdx2^NSE-FlpO^* we saw widespread expression across the cortex and forebrain (Figure 4G). *Cdx2^Cre^* mice were generated by combining a 9.5 kb promoter fragment of the human *Cdx2* gene, a Cre sequence that contained a neural specific enhancer sequence, and a 360 bp human beta-actin polyadenylation cassette (Hinoi et al., 2007). The ectopic expression observed in *Cdx2^Cre^*;LSL-TdTomato tissue could potentially be a result of germline recombination of the LSL-TdTomato during development of the neuroectoderm, resulting in unexpected brain expression in the adult. In spite of these caveats, these lines are still valuable tools in the study of pain and itch. However, the following recommendations should be followed: when using the *Cdx2^NSE-FlpO^*, it is advised to check for germline recombination in tail DNA, which will help determine which animals to exclude for behavioral analysis. For both *Cdx2^NSE-FlpO^* and *Cdx2^Cre^*, it is advisable to cross breeder males to reporter females and check the brain for levels of germline recombination, specifically in the cortex and forebrain as observed in this study. Additionally, intersectional approaches should have built-in reporters when possible, so that brain expression can be assessed. For example, one could use an LSL-TdTomato reporter in a Cdx2Cre;Gene-flox cross, allowing one to visualize if Cre-mediated LSL-TdTomato expression is present in the brain. Lastly, in the absence of a reporter, tissue can be genotyped following behavioral experiments, and can be checked for brain recombination of Cre-dependent transgene expression. In addition to the *Cdx2^NSE-FlpO^* used in this study, there is another *Cdx2^FlpO^* mouse line (Britz et al., 2015) available that utilizes a similar design to the *Cdx2^Cre^*. Given the brain recombination observed in the *Cdx2^Cre^* (Figure 4H; Supplementary Figure S1I), we recommend similar precautions when using this or any other caudal-targeting mouse line for brain-sparing manipulations.

In addition to the *Hoxb8^FlpO^*, there are two *Hoxb8^Cre^* mouse lines (Chen et al., 2010; Witschi et al., 2010) that also use the *Hoxb8* gene to achieve brain-sparing targeting. The first mouse line uses a targeted knockin approach where an Internal Ribosome Entry Site or IRES followed by a Cre recombinase were knocked into the 3′ UnTranslated Region (UTR) of the gene. This strategy is not expected to disrupt endogenous gene expression. However translational efficiency of the IRES-dependent second gene is usually around 20–50% that of the first gene (Mizuguchi et al., 2000), which suggests that Cre expression levels might not always be sufficient to induce Cre recombination in all *Hoxb8+* cells. The second mouse line utilized a 11.4 kb sequence upstream of the *Hoxb8* gene, which included most of *Hoxb9* as well as the first 1,058 bp of *Hoxb8*, and fused it to a 35 bp sequence containing a Kozak sequence and a start ATG (Witschi et al., 2010). A *Cre* recombinase cassette and a poly(A) sequence were inserted downstream of the ATG and the construct was injected in the pronucleus to create a transgenic mouse line. Although the generation of transgenic mouse lines has its drawbacks due to the random insertion of the transgene in the genome and the potential for disruption of other genes, this strategy should not disrupt normal endogenous *Hoxb8* gene expression. In the generation of the *Hoxb8^FlpO^* mouse line, we utilized a T2A sequence fused to a FlpO sequence, which was knocked in just before the stop codon of *Hoxb8* exon 2. This approach has an advantage due to the self-cleaving ability of the T2A sequence, which results in functional FlpO recombinase function without influencing endogenous *Hoxb8* function. Notably the T2A sequence usually has a cleavage efficiency close to 100% meaning stoichiometric expression of the two genes flanking the peptide (Kim et al., 2011). Since the *Hoxb8^FlpO^* was generated as a knock-in using CRISPR-Cas9 technology, we do not expect the background-dependent effects on expression profile observed with other lines, nor do we expect any disruption of endogenous *Hoxb8* function. Furthermore, we did not observe any germline recombination in *Hoxb8^FlpO^* brain tissue. While we observed no neuronal TdTomato expression in *Hoxb8^FlpO^* brain tissue, we did observe TdTomato fluorescence in microglia, which is consistent with the expression pattern of *Hoxb8* in mice. Since *Hoxb8*-lineage microglia account for one-third of all adult microglia in the mouse (De et al., 2018), it is expected to see *Hoxb8*+ microglia in the cortex. Lastly, a key factor for the utilization of *Hoxb8^FlpO^* mice in studying somatosensation is to ensure that insertion of a FlpO sequence into the *Hoxb8* locus does not alter sensory responses or motor performance. Here, we show that heterozygous and homozygous *Hoxb8^FlpO^* male and female mice exhibit none of the phenotypes associated with *Hoxb8* mutants, including abnormal laminar organization of the dorsal horn, heat and mechanical sensory deficits (Holstege et al., 2008), excessive grooming (Chen et al., 2010), or aberrant motor behavior (van den Akker et al., 1999). Collectively, the *Hoxb8^FlpO^* mouse line represents a useful tool for the study of pain and itch because it offers the ability to restrict manipulations to the spinal cord and DRG.

Although invaluable, intersectional genetics can be very costly, requiring time for complex matings as well as high mouse colony costs for the breeding and maintenance of various recombinase-dependent reporters. Here we describe a strategy using a Cre-DOG virus and intersectional GFP expression (Figures 6, 7; Discussion Figure) utilized in a modular way to incorporate any available Cre-dependent viral strategies, greatly reducing the cage costs associated with mouse genetics. Additionally, this approach is useful in manipulating neurons in the adult mouse which are defined by a molecular lineage during development, especially in cases of transient gene expression where Cre expression is not present in the adult animal. While this study utilizes Cre-DOG to drive expression of a chemogenetic virus (Figure 7), one could also utilize a Cre-dependent channelrhodopsin virus for optogenetic control of this population, or a Cre-dependent calcium indicator virus for calcium imaging, among other applications. Therefore, this approach allows for genetic access to a developmentally determined intersection through GFP expression, with the added benefit of modular usage of any available Cre-dependent viruses. Another utilization of this approach is to use *CreER* mice (Feil et al., 1996; Indra et al., 1999), where the expression of GFP can be induced and restricted to a specific developmental time point. This strategy would allow for the comparison of early vs. late developmental populations using a Cre-DOG virus to leverage time-locked GFP expression for functional studies. Furthermore, there are over 1,000 existing transgenic GFP mouse lines that have been well characterized (Gong et al., 2003; Heintz, 2004). Rather than generating and validating Cre or Flp lines for manipulating these populations, one could instead utilize Cre-DOG to manipulate already characterized GFP lines. This strategy is much more accessible than traditional intersectional genetic approaches which require the long-term maintenance of multiple transgenic recombinase and reporter lines (Tang et al., 2015). One point of caution regarding the use of viral approaches concerns the inherent variability in levels of infection, which can be assessed by post-hoc histological analysis. We identify here an interesting intermediate locomotor phenotype when targeting adult (ad-*Ror𝛽*) vs. adult + developmental (ad + dev-*Ror𝛽*) *Ror𝛽+ via* local viral injection compared to developmental whole-body knock out of the *Ror𝛽* gene. While our approach has several advantages listed above, including circumventing developmental plasticity-related genetic compensation, the identified intermediate phenotype could be in part due to incomplete silencing of the *Ror𝛽* population.

In summary, our *Hoxb8^FlpO^* mouse line is compatible with intersectional Cre-Flp approaches and can be utilized to target spinal and DRG neurons to study mechanisms of nociception, touch (Abraira and Ginty, 2013; Abraira et al., 2017), itch (Koch et al., 2018; Chen and Sun, 2020), proprioception (Johnson et al., 2008; Takeoka and Arber, 2019), and locomotion (Côté et al., 2018). We anticipate that this mouse line will be useful in elucidating spinal versus supraspinal effects of genes with widespread expression patterns. Furthermore, we describe here an approach using viral Cre-DOG technology to target developmentally determined neuronal populations in the adult. Collectively, these tools will allow for the dissection of spinal versus supraspinal contributions to sensory and motor function, as well as the comparison and manipulation of developmentally determined adult lineages.

## Data availability statement

The original contributions presented in the study are included in the article/Supplementary material, further inquiries can be directed to the corresponding author.

## Ethics statement

The animal study was reviewed and approved by Rutgers IACUC; protocol #: 201702589.

## Author contributions

MB, AU, JK, RS, BAB, CA, BNB, MR, MG, and GS performed histological experiments and analysis. MB, AU, and JE performed behavioral experiments and analysis. HN, OO, and AM helped with kinematics analysis. PR led the generation of the Hoxb8FlpO mouse line. MB, AU, JK, and JE prepared the figures with the contribution of all the authors. VA, MB, and AU conceived and supervised the study. AU and MB wrote the paper with help from VA. All authors contributed to the article and approved the submitted version.

## Funding

Financial support was provided by Pew Charitable Trust (VA); NIH/NINDS R01NS119268 (VA), New Jersey Commission on Spinal Cord Research (VA), and Craig H. Neilsen Foundation (VA), F32MH127772 (JE).

## Conflict of interest

The authors declare that the research was conducted in the absence of any commercial or financial relationships that could be construed as a potential conflict of interest.

## Publisher’s note

All claims expressed in this article are solely those of the authors and do not necessarily represent those of their affiliated organizations, or those of the publisher, the editors and the reviewers. Any product that may be evaluated in this article, or claim that may be made by its manufacturer, is not guaranteed or endorsed by the publisher.

## Footnotes

^1^http://mousebrain.org/adolescent/genesearch.html

## Supplementary material

SUPPLEMENTARY FIGURE S1 *Hoxb8^FlpO^* expression is present in the DRG but not in neurons of the brain, *Cdx2^Cre^* expression can be observed in neurons of the brain. **(A)** Zoomed in image of a transverse lumbar spinal cord section from an adult *Hoxb8^FlpO^*;FSF-TdTomato mouse, showing Hoxb8FlpO expression is not present in spinal microglia. *Hoxb8^FlpO^*;FSF-TdTomato fluorescence (red) is not colocalized with Iba1+ microglia (blue). **(B,C)** Images of cryosectioned cervical **(B)** and thoracic **(C)** DRG from an adult *Hoxb8^FlpO^*;FSF-TdTomato mouse. *Hoxb8^FlpO^*;FSF-TdTomato fluorescence (red) is colocalized with the vast majority of DRG neurons, including CGRP+ (green) and IB4+ (blue) DRG neurons. **(D,E)** Images of cryosectioned cervical **(D)** and thoracic **(E)** DRG from an adult *Hoxb8^FlpO^*;FSF-TdTomato mouse. *Hoxb8^FlpO^*;FSF-TdTomato fluorescence (red) is colocalized with the large diameter DRG neuron marker NFH (green).

SUPPLEMENTARY FIGURE S2 *Hoxb8^FlpO^* expression is present in caudal viscera and brain microglia, but not in neurons of the brain. *Cdx2^Cre^* expression can be observed in neurons of the brain. **(A)** Image of cryosectioned hindpaw glabrous skin from an adult *Hoxb8^FlpO^*;FSF-TdTomato mouse, showing TdTomato expression (red) in the subcutis, with very sparse labeling in the reticular dermis. DAPI (blue) was used as a counterstain. **(B)** Image of cryosectioned kidney from an adult *Hoxb8^FlpO^*;FSF-TdTomato mouse, showing TdTomato expression (red) in endothelial cells. DAPI (blue) was used as a counterstain. **(C)** Image of cryosectioned liver from an adult *Hoxb8^FlpO^*;FSF-TdTomato mouse, showing an absence of TdTomato fluorescence in liver tissue. DAPI (blue) was used as a counterstain. **(D)** Image of cryosectioned heart from an adult *Hoxb8^FlpO^*;FSF-TdTomato mouse, showing an absence of TdTomato fluorescence in cardiac tissue. DAPI (blue) was used as a counterstain. **(E)** Zoomed in image of the cortex in coronal brain tissue from an adult *Hoxb8^FlpO^*;FSF-TdTomato mouse, showing the presence of TdTomato+ microglia (red) that are not colocalized with NeuN+ neurons (green). **(F,G)** Image of coronal brain and brainstem from adult *Cdx2^Cre^*;LSL-TdTomato mice, showing neuronal TdTomato fluorescence (red) in both the somatosensory cortex **(F)** and parabrachial nucleus **(G)** **(H,I,J)** Images of sagittal sections of caudal cortex from an adult *Hoxb8^FlpO^*;FSF-TdTomato mouse on a mixed genetic background (C57BL/6/FVB) **(H)** and from adult *Cdx2^NSE-FlpO^*; FSF-TdTomato mice on a 25% FVB background **(I)** and a 50% FVB background **(J)** showing TdTomato fluorescence (red) in neurons (NeuN in green) in the brain of *Cdx2^NSE-FlpO^* but not *Hoxb8^FlpO^* mice. We never observed brain neuronal expression in *Hoxb8^FlpO^*;FSF-TdTomato mice, independent of background.

SUPPLEMENTARY FIGURE S3 Homozygous and heterozygous *Hoxb8^FlpO^* mice have normal locomotor gait **(A)** *Hoxb8^FlpO^* and *Hoxb8*^*FlpO/*+^ mice have normal stride length (cm) across all limbs compared to wild type controls. Data is represented as mean+individual data points (n = 9-15 mice per group), and significance was assessed using a one-way ANOVA with Tukey’s multiple comparisons. **(B)** *Hoxb8^FlpO^* and *Hoxb8*^*FlpO/*+^ mice have normal stride duration (s) across all limbs compared to wild type controls. Data is represented as mean+individual data points (n = 9-15 mice per group), and significance was assessed using a one-way ANOVA with Tukey’s multiple comparisons.

SUPPLEMENTARY FIGURE S4 The viral and transgenic intersectional strategy can target adult or adult & developmental Rorβ spinal neuronal populations. **(A)** Viral control: a *Ror𝛽^Cre^*;*Hoxb8^FlpO^*; RC::FLTG mouse was injected with Cre-DOG and AAV-LSL-GFP viruses. An image of a transverse section of the spinal cord shows Cre-DOG-dependent GFP expression at the site of injection. **(B)** Adult Rorβ spinal neuronal population silencing: a *Ror𝛽^Cre^*; RC::FLTG mouse was injected with Cre-DOG and AAV-LSL-Di-mCherry, targeting the adult Rorβ spinal neuronal population that still expresses Rorβ. An image of a transverse section of the spinal cord shows Cre-DOG-dependent Di-mCherry expression (red) in neural processes. **(C)** Adult & developmental Rorβ spinal neuronal population silencing: a *Ror𝛽^Cre^*;*Hoxb8^FlpO^*;RC::FLTG mouse was injected with Cre-DOG and AAV-LSL-Di-mCherry, targeting the adult & developmental Rorβ spinal neuronal population targeted by the *Ror𝛽^Cre^*;*Hoxb8^FlpO^*; RC::FLTG intersection. An image of a transverse section of the spinal cord shows Cre-DOG-dependent Di-mCherry expression (red).

## References

[ref1] Abraira V. E. Ginty D. D. (2013). The sensory neurons of touch. Neuron 79, 618–639. doi: 10.1016/j.neuron.2013.07.051, PMID: 23972592 PMC3811145

[ref2] Abraira V. E. Kuehn E. D. Chirila A. M. Springel M. W. Toliver A. A. Zimmerman A. L.. (2017). The cellular and synaptic architecture of the Mechanosensory dorsal horn. Cells 168, 295–310.e19. doi: 10.1016/j.cell.2016.12.010, PMID: 28041852 PMC5236062

[ref3] Adrienne E. D. Ardem P. (2010). Nociceptors: the sensors of the pain pathway. J. Clin. Invest. 120, 3760–3772. doi: 10.1172/JCI42843, PMID: 21041958 PMC2964977

[ref4] Awatramani R. Soriano P. Rodriguez C. Mai J. J. Dymecki S. M. (2003). Cryptic boundaries in roof plate and choroid plexus identified by intersectional gene activation. Nat. Genet. 35, 70–75. doi: 10.1038/ng1228, PMID: 12923530

[ref5] Bang S. J. Jensen P. Dymecki S. M. Commons K. G. (2012). Projections and interconnections of genetically defined serotonin neurons in mice. Eur. J. Neurosci. 35, 85–96. doi: 10.1111/j.1460-9568.2011.07936.x, PMID: 22151329 PMC3268345

[ref6] Barik A. Sathyamurthy A. Thompson J. Seltzer M. Levine A. Chesler A. (2021). A spinoparabrachial circuit defined by Tacr1 expression drives pain. elife 10. doi: 10.7554/eLife.61135 PMC7993995 33591273

[ref7] Boinon L. Yu J. Madura C. L. Chefdeville A. Feinstein D. L. Moutal A.. (2022). Conditional knockout of CRMP2 in neurons, but not astrocytes, disrupts spinal nociceptive neurotransmission to control the initiation and maintenance of chronic neuropathic pain. Pain 163, e368–e381. doi: 10.1097/j.pain.0000000000002344, PMID: 35029600 PMC8760468

[ref8] Bourane S. Duan B. Koch S. C. Dalet A. Britz O. Garcia-Campmany L.. (2015a). Gate control of mechanical itch by a subpopulation of spinal cord interneurons. Science 350, 550–554. doi: 10.1126/science.aac8653, PMID: 26516282 PMC4700934

[ref9] Bourane S. Grossmann K. S. Britz O. Dalet A. Del Barrio M. G. Stam F. J.. (2015b). Identification of a spinal circuit for light touch and fine motor control. Cells 160, 503–515. doi: 10.1016/j.cell.2015.01.011, PMID: 25635458 PMC4431637

[ref10] Brian Roome R. Bourojeni F. B. Mona B. Rastegar-Pouyani S. Blain R. Dumouchel A.. (2020). Phox2a defines a developmental origin of the anterolateral system in mice and humans. Cell Rep. 33:108425. doi: 10.1016/j.celrep.2020.108425, PMID: 33238113 PMC7713706

[ref11] Britz O. Zhang J. Grossmann K. S. Dyck J. Kim J. C. Dymecki S.. (2015). A genetically defined asymmetry underlies the inhibitory control of flexor-extensor locomotor movements. elife 4. doi: 10.7554/eLife.04718, PMID: 26465208 PMC4604447

[ref12] Buch T. Heppner F. L. Tertilt C. Heinen T. J. A. J. Kremer M. Wunderlich F. T.. (2005). A Cre-inducible diphtheria toxin receptor mediates cell lineage ablation after toxin administration. Nat. Methods 2, 419–426. doi: 10.1038/nmeth762 15908920

[ref13] Butler S. J. Bronner M. E. (2015). From classical to current: analyzing peripheral nervous system and spinal cord lineage and fate. Dev. Biol. 398, 135–146. doi: 10.1016/j.ydbio.2014.09.033, PMID: 25446276 PMC4845735

[ref14] Catela C. Chen Y. Weng Y. Wen K. Kratsios P. (2022). Control of spinal motor neuron terminal differentiation through sustained Hoxc8 gene activity. elife 11. doi: 10.7554/elife.70766, PMID: 35315772 PMC8940177

[ref15] Chaplan S. R. Bach F. W. Pogrel J. W. Chung J. M. Yaksh T. L. (1994). Quantitative assessment of tactile allodynia in the rat paw. J. Neurosci. Methods 53, 55–63. doi: 10.1016/0165-0270(94)90144-9 7990513

[ref16] Charité J. de Graaff W. Vogels R. Meijlink F. Deschamps J. (1995). Regulation of the Hoxb-8 gene: synergism between multimerized cis-acting elements increases responsiveness to positional information. Dev. Biol. 171, 294–305. doi: 10.1006/dbio.1995.1282, PMID: 7556914

[ref17] Chen L. Huang J. Zhao P. Persson A.-K. Dib-Hajj F. B. Cheng X.. (2018). Conditional knockout of NaV1.6 in adult mice ameliorates neuropathic pain. Sci. Rep. 8:3845. doi: 10.1038/s41598-018-22216-w, PMID: 29497094 PMC5832877

[ref18] Chen X.-J. Sun Y.-G. (2020). Central circuit mechanisms of itch. Nat. Commun. 11:3052. doi: 10.1038/s41467-020-16859-5, PMID: 32546780 PMC7297978

[ref19] Chen S.-K. Tvrdik P. Peden E. Cho S. Wu S. Spangrude G.. (2010). Hematopoietic origin of pathological grooming in Hoxb8 mutant mice. Cells 141, 775–785. doi: 10.1016/j.cell.2010.03.055, PMID: 20510925 PMC2894573

[ref20] Chirila A. M. Rankin G. Tseng S.-Y. Emanuel A. J. Chavez-Martinez C. L. Zhang D.. (2022). Mechanoreceptor signal convergence and transformation in the dorsal horn flexibly shape a diversity of outputs to the brain. Cells 185, 4541–4559.e23. doi: 10.1016/j.cell.2022.10.012, PMID: 36334588 PMC9691598

[ref21] Choi S. Hachisuka J. Brett M. A. Magee A. R. Omori Y. Iqbal N.-U.-A.. (2020). Parallel ascending spinal pathways for affective touch and pain. Nature 587, 258–263. doi: 10.1038/s41586-020-2860-1, PMID: 33116307 PMC7666110

[ref22] Côté M.-P. Murray L. M. Knikou M. (2018). Spinal control of locomotion: individual neurons, their circuits and functions. Front. Physiol. 9:784. doi: 10.3389/fphys.2018.00784, PMID: 29988534 PMC6026662

[ref23] Coutaud B. Pilon N. (2013). Characterization of a novel transgenic mouse line expressing Cre recombinase under the control of the Cdx2 neural specific enhancer. Genesis 51, 777–784. doi: 10.1002/dvg.22421, PMID: 23913642

[ref24] Cui L. Miao X. Liang L. Abdus-Saboor I. Olson W. Fleming M. S.. (2016). Identification of early RET+ deep dorsal spinal cord interneurons in gating pain. Neuron 91, 1137–1153. doi: 10.1016/j.neuron.2016.07.038, PMID: 27545714 PMC5017914

[ref25] Daigle T. L. Madisen L. Hage T. A. Valley M. T. Knoblich U. Larsen R. S.. (2018). A suite of transgenic driver and reporter mouse lines with enhanced brain-cell-type targeting and functionality. Cells 174, 465–480.e22. doi: 10.1016/j.cell.2018.06.035, PMID: 30007418 PMC6086366

[ref26] De S. Van Deren D. Peden E. Hockin M. Boulet A. Titen S.. (2018). Two distinct ontogenies confer heterogeneity to mouse brain microglia. Development 145. doi: 10.1242/dev.152306, PMID: 29973370 PMC6053660

[ref27] Deschamps J. Wijgerde M. (1993). Two phases in the establishment of HOX expression domains. Dev. Biol. 156, 473–480. doi: 10.1006/dbio.1993.1093, PMID: 8096483

[ref28] Ding Y.-Q. Yin J. Kania A. Zhao Z.-Q. Johnson R. L. Chen Z.-F. (2004). Lmx1b controls the differentiation and migration of the superficial dorsal horn neurons of the spinal cord. Development 131, 3693–3703. doi: 10.1242/dev.01250, PMID: 15229182

[ref30] Dorman C. W. Krug H. E. Frizelle S. P. Funkenbusch S. Mahowald M. L. (2014). A comparison of DigiGait™ and TreadScan™ imaging systems: assessment of pain using gait analysis in murine monoarthritis. J. Pain Res. 7, 25–35. doi: 10.2147/JPR.S52195, PMID: 24516338 PMC3883276

[ref31] Duan B. Cheng L. Bourane S. Britz O. Padilla C. Garcia-Campmany L.. (2014). Identification of spinal circuits transmitting and gating mechanical pain. Cells 159, 1417–1432. doi: 10.1016/j.cell.2014.11.003, PMID: 25467445 PMC4258511

[ref32] Dymecki S. M. Ray R. S. Kim J. C. (2010). Mapping cell fate and function using recombinase-based intersectional strategies. Methods Enzymol. 477, 183–213. doi: 10.1016/S0076-6879(10)77011-7, PMID: 20699143

[ref33] Eisdorfer J. T. Sobotka-Briner H. Schramfield S. Moukarzel G. Chen J. Campion T. J.. (2022). Chemogenetic modulation of sensory afferents induces locomotor changes and plasticity after spinal cord injury. Front. Mol. Neurosci. 15:872634. doi: 10.3389/fnmol.2022.872634, PMID: 36090254 PMC9461563

[ref34] Escalante A. Klein R. (2020). Spinal inhibitory Ptf1a-derived neurons prevent self-generated itch. Cell Rep. 33:108422. doi: 10.1016/j.celrep.2020.108422, PMID: 33238109

[ref35] Farago A. F. Awatramani R. B. Dymecki S. M. (2006). Assembly of the brainstem cochlear nuclear complex is revealed by intersectional and subtractive genetic fate maps. Neuron 50, 205–218. doi: 10.1016/j.neuron.2006.03.014, PMID: 16630833

[ref36] Feil R. Brocard J. Mascrez B. LeMeur M. Metzger D. Chambon P. (1996). Ligand-activated site-specific recombination in mice. Proc. Natl. Acad. Sci. U. S. A. 93, 10887–10890. doi: 10.1073/pnas.93.20.10887, PMID: 8855277 PMC38252

[ref37] Fenno L. E. Ramakrishnan C. Kim Y. S. Evans K. E. Lo M. Vesuna S.. (2020). Comprehensive dual- and triple-feature intersectional single-vector delivery of diverse functional payloads to cells of behaving mammals. Neuron 107, 836–853.e11. doi: 10.1016/j.neuron.2020.06.003, PMID: 32574559 PMC7687746

[ref38] Fiander M. D. Chedrawe M. A. Lamport A.-C. Akay T. Robertson G. S. (2017). Sagittal plane kinematic gait analysis in C57BL/6 mice subjected to MOG35-55 induced experimental autoimmune encephalomyelitis. J. Vis. Exp. doi: 10.3791/56032, PMID: 29155752 PMC5755292

[ref39] Gingras J. Smith S. Matson D. J. Johnson D. Nye K. Couture L.. (2014). Global Nav1.7 knockout mice recapitulate the phenotype of human congenital indifference to pain. PLoS One 9:e105895. doi: 10.1371/journal.pone.0105895, PMID: 25188265 PMC4154897

[ref40] Gong S. Zheng C. Doughty M. L. Losos K. Didkovsky N. Schambra U. B.. (2003). A gene expression atlas of the central nervous system based on bacterial artificial chromosomes. Nature 425, 917–925. doi: 10.1038/nature02033, PMID: 14586460

[ref41] Graham A. Maden M. Krumlauf R. (1991). The murine Hox-2 genes display dynamic dorsoventral patterns of expression during central nervous system development. Development 112, 255–264. doi: 10.1242/dev.112.1.255, PMID: 1685115

[ref42] Greer J. M. Capecchi M. R. (2002). Hoxb8 is required for normal grooming behavior in mice. Neuron 33, 23–34. doi: 10.1016/S0896-6273(01)00564-5 11779477

[ref43] Gross M. K. Dottori M. Goulding M. (2002). Lbx1 specifies somatosensory association interneurons in the dorsal spinal cord. Neuron 34, 535–549. doi: 10.1016/S0896-6273(02)00690-6, PMID: 12062038

[ref44] Harris J. A. Hirokawa K. E. Sorensen S. A. Gu H. Mills M. Ng L. L.. (2014). Anatomical characterization of Cre driver mice for neural circuit mapping and manipulation. Front. Neural Circuits 8:76. doi: 10.3389/fncir.2014.00076 25071457 PMC4091307

[ref005] He E. Zhang X. Ren S. Sun J. (2016). Deep residual learning for image recognition. In Proceedings of the IEEE conference on computer vision and pattern recognition, 770–778. Available at: https://arxiv.org/abs/1512.03385.

[ref45] Heintz N. (2004). Gene expression nervous system atlas (GENSAT). Nat. Neurosci. 7:483. doi: 10.1038/nn0504-483, PMID: 15114362

[ref46] Hinoi T. Akyol A. Theisen B. K. Ferguson D. O. Greenson J. K. Williams B. O.. (2007). Mouse model of colonic adenoma-carcinoma progression based on somatic Apc inactivation. Cancer Res. 67, 9721–9730. doi: 10.1158/0008-5472.CAN-07-2735, PMID: 17942902

[ref47] Holstege J. C. de Graaff W. Hossaini M. Cano S. C. Jaarsma D. van den Akker E.. (2008). Loss of Hoxb8 alters spinal dorsal laminae and sensory responses in mice. Proc. Natl. Acad. Sci. U. S. A. 105, 6338–6343. doi: 10.1073/pnas.0802176105, PMID: 18430798 PMC2359815

[ref48] Hooks B. M. Lin J. Y. Guo C. Svoboda K. (2015). Dual-channel circuit mapping reveals sensorimotor convergence in the primary motor cortex. J. Neurosci. 35, 4418–4426. doi: 10.1523/JNEUROSCI.3741-14.2015, PMID: 25762684 PMC4355205

[ref49] Huang T. Lin S.-H. Malewicz N. M. Zhang Y. Zhang Y. Goulding M.. (2018). Identifying the pathways required for coping behaviours associated with sustained pain. Nature 565, 86–90. doi: 10.1038/s41586-018-0793-8 30532001 PMC6461409

[ref50] Hughes D. I. Sikander S. Kinnon C. M. Boyle K. A. Watanabe M. Callister R. J.. (2012). Morphological, neurochemical and electrophysiological features of parvalbumin-expressing cells: a likely source of axo-axonic inputs in the mouse spinal dorsal horn. J. Physiol. 590, 3927–3951. doi: 10.1113/jphysiol.2012.235655, PMID: 22674718 PMC3476641

[ref51] Indra A. K. Warot X. Brocard J. Bornert J.-M. Xiao J.-H. Chambon P.. (1999). Temporally-controlled site-specific mutagenesis in the basal layer of the epidermis: comparison of the recombinase activity of the tamoxifen-inducible Cre-ERT and Cre-ERT2 recombinases. Nucleic Acids Res. 27, 4324–4327. doi: 10.1093/nar/27.22.4324, PMID: 10536138 PMC148712

[ref006] Insafutdinov E. Pishchulin L. Andres B. Andriluka M. Schiele B. (2016). DeeperCut: a deeper, stronger, and faster multi-person pose estimation model. European Conference on Computer Vision 34–50.

[ref52] Jessell T. M. (2000). Neuronal specification in the spinal cord: inductive signals and transcriptional codes. Nat. Rev. Genet. 1, 20–29. doi: 10.1038/35049541 11262869

[ref53] Johnson E. O. Babis G. C. Soultanis K. C. Soucacos P. N. (2008). Functional neuroanatomy of proprioception. J. Surg. Orthop. Adv. 17, 159–164. PMID: 18851800

[ref54] Kakava-Georgiadou N. Zwartkruis M. M. Bullich-Vilarrubias C. Luijendijk M. C. M. Garner K. M. van der Plasse G.. (2019). An intersectional approach to target neural circuits with cell- and projection-type specificity: validation in the mesolimbic dopamine system. Front. Mol. Neurosci. 12:49. doi: 10.3389/fnmol.2019.00049, PMID: 30873002 PMC6403677

[ref55] Kim J. H. Lee S.-R. Li L.-H. Park H.-J. Park J.-H. Lee K. Y.. (2011). High cleavage efficiency of a 2A peptide derived from porcine teschovirus-1 in human cell lines, zebrafish and mice. PLoS One 6:e18556. doi: 10.1371/journal.pone.0018556, PMID: 21602908 PMC3084703

[ref56] Knockout Mice Fact Sheet (2022). Genome.gov. Available at: https://www.genome.gov/about-genomics/fact-sheets/Knockout-Mice-Fact-Sheet (Accessed April 24, 2022).

[ref57] Koch S. C. Acton D. Goulding M. (2018). Spinal circuits for touch, pain, and itch. Annu. Rev. Physiol. 80, 189–217. doi: 10.1146/annurev-physiol-022516-034303, PMID: 28961064 PMC5891508

[ref58] Koch S. C. Del Barrio M. G. Dalet A. Gatto G. Gunther T. Zhang J.. (2017). RORβ spinal interneurons gate sensory transmission during locomotion to secure a fluid walking gait. Neuron 96, 1419–1431.e5. doi: 10.1016/j.neuron.2017.11.011, PMID: 29224725 PMC5828033

[ref59] Lai H. C. Seal R. P. Johnson J. E. (2016). Making sense out of spinal cord somatosensory development. Development 143, 3434–3448. doi: 10.1242/dev.139592, PMID: 27702783 PMC5087618

[ref60] Lau J. Minett M. S. Zhao J. Dennehy U. Wang F. Wood J. N.. (2011). Temporal control of gene deletion in sensory ganglia using a tamoxifen-inducible Advillin-Cre-ERT2 recombinase mouse. Mol. Pain 7:100. 22188729 10.1186/1744-8069-7-100 PMC3260248

[ref61] Lee S. K. Pfaff S. L. (2001). Transcriptional networks regulating neuronal identity in the developing spinal cord. Nat. Neurosci. 4, 1183–1191. doi: 10.1038/nn750, PMID: 11687828

[ref62] Lu D. C. Niu T. Alaynick W. A. (2015). Molecular and cellular development of spinal cord locomotor circuitry. Front. Mol. Neurosci. 8:25. doi: 10.3389/fnmol.2015.00025 26136656 PMC4468382

[ref63] Madisen L. Garner A. R. Shimaoka D. Chuong A. S. Klapoetke N. C. Li L.. (2015). Transgenic mice for intersectional targeting of neural sensors and effectors with high specificity and performance. Neuron 85, 942–958. doi: 10.1016/j.neuron.2015.02.022, PMID: 25741722 PMC4365051

[ref64] Madisen L. Mao T. Koch H. Zhuo J.-M. Berenyi A. Fujisawa S.. (2012). A toolbox of Cre-dependent optogenetic transgenic mice for light-induced activation and silencing. Nat. Neurosci. 15, 793–802. doi: 10.1038/nn.3078, PMID: 22446880 PMC3337962

[ref65] Madisen L. Zwingman T. A. Sunkin S. M. Oh S. W. Zariwala H. A. Gu H.. (2009). A robust and high-throughput Cre reporting and characterization system for the whole mouse brain. Nat. Neurosci. 13, 133–140. doi: 10.1038/nn.2467 20023653 PMC2840225

[ref66] Masgutova G. Harris A. Jacob B. Corcoran L. M. Clotman F. (2019). Pou2f2 regulates the distribution of dorsal interneurons in the mouse developing spinal cord. Front. Mol. Neurosci. 12:263. doi: 10.3389/fnmol.2019.00263, PMID: 31787878 PMC6853997

[ref67] Matthaei K. I. (2007). Genetically manipulated mice: a powerful tool with unsuspected caveats. J. Physiol. 582, 481–488. doi: 10.1113/jphysiol.2007.134908 17495035 PMC2075346

[ref003] Mathis A. Mamidanna P. Cury K. M Abe T. Murthy V. N. Mathis M. W.. (2018). Deeplabcut: markerless pose estimation of user-defined body parts with deep learning. Nature Neuroscience. 21, 1281–1289. 30127430 10.1038/s41593-018-0209-y

[ref68] Mazzoni E. O. Mahony S. Peljto M. Patel T. Thornton S. R. McCuine S.. (2013). Saltatory remodeling of Hox chromatin in response to rostrocaudal patterning signals. Nat. Neurosci. 16, 1191–1198. doi: 10.1038/nn.3490, PMID: 23955559 PMC3799941

[ref69] Meltzer S. Boulanger K. C. Chirila A. M. Osei-Asante E. DeLisle M. Zhang Q.. (2023). γ-Protocadherins control synapse formation and peripheral branching of touch sensory neurons. Neuron. doi: 10.1016/j.neuron.2023.03.012 PMC10365546 37028432

[ref70] Metzis V. Steinhauser S. Pakanavicius E. Gouti M. Stamataki D. Ivanovitch K.. (2018). Nervous system regionalization entails axial allocation before neural differentiation. Cells 175, 1105–1118.e17. doi: 10.1016/j.cell.2018.09.040, PMID: 30343898 PMC6218657

[ref71] Mills E. P. Di Pietro F. Alshelh Z. Peck C. C. Murray G. M. Vickers E. R.. (2018). Brainstem pain-control circuitry connectivity in chronic neuropathic pain. J. Neurosci. 38, 465–473. doi: 10.1523/JNEUROSCI.1647-17.2017, PMID: 29175957 PMC6596109

[ref72] Mizuguchi H. Xu Z. Ishii-Watabe A. Uchida E. Hayakawa T. (2000). IRES-dependent second gene expression is significantly lower than cap-dependent first gene expression in a Bicistronic vector. Mol. Ther. 1, 376–382. doi: 10.1006/mthe.2000.0050, PMID: 10933956

[ref73] Nagarajan N. Jones B. W. West P. J. Marc R. E. Capecchi M. R. (2017). Corticostriatal circuit defects in Hoxb8 mutant mice. Mol. Psychiatry 23, 1868–1877. doi: 10.1038/mp.2017.180 28948967 PMC5970001

[ref004] Nath T. Mathis A. Chen A. C. Patel A. Bethge M. Mathis M. W. (2019). Using deeplabcut for 3d markerless pose estimation across species and behaviors. Nature Protocols 14, 2152–2176., PMID: 31227823 10.1038/s41596-019-0176-0

[ref74] Niederkofler V. Asher T. E. Okaty B. W. Rood B. D. Narayan A. Hwa L. S.. (2016). Identification of serotonergic neuronal modules that affect aggressive behavior. Cell Rep. 17, 1934–1949. doi: 10.1016/j.celrep.2016.10.063, PMID: 27851959 PMC5156533

[ref75] Orefice L. L. Mosko J. R. Morency D. T. Wells M. F. Tasnim A. Mozeika S. M.. (2019). Targeting peripheral somatosensory neurons to improve tactile-related phenotypes in ASD models. Cells 178, 867–886.e24. doi: 10.1016/j.cell.2019.07.024, PMID: 31398341 PMC6704376

[ref76] Orefice L. L. Zimmerman A. L. Chirila A. M. Sleboda S. J. Head J. P. Ginty D. D. (2016). Peripheral Mechanosensory neuron dysfunction underlies tactile and behavioral deficits in mouse models of ASDs. Cells 166, 299–313. doi: 10.1016/j.cell.2016.05.033, PMID: 27293187 PMC5567792

[ref77] Paixão S. Loschek L. Gaitanos L. Morales P. A. Goulding M. Klein R. (2019). Identification of spinal neurons contributing to the dorsal column projection mediating fine touch and corrective motor movements. Neuron 104, 749–764.e6. doi: 10.1016/j.neuron.2019.08.029, PMID: 31586516 PMC7814917

[ref78] Pan H. Fatima M. Li A. Lee H. Cai W. Horwitz L.. (2019). Identification of a spinal circuit for mechanical and persistent spontaneous itch. Neuron 103, 1135–1149.e6. doi: 10.1016/j.neuron.2019.06.016, PMID: 31324538 PMC6763390

[ref79] Parkitna J. R. Engblom D. Schütz G. (2009). Generation of Cre recombinase-expressing transgenic mice using bacterial artificial chromosomes. Methods Mol. Biol. 530, 325–342. doi: 10.1007/978-1-59745-471-1_17, PMID: 19266340

[ref80] Peirs C. Williams S. G. Zhao X. Arokiaraj C. M. Ferreira D. W. Noh M. C.. (2021). Mechanical allodynia circuitry in the dorsal horn is defined by the nature of the injury. Neuron 109, 73–90.e7. doi: 10.1016/j.neuron.2020.10.027, PMID: 33181066 PMC7806207

[ref81] Peirs C. Williams S.-P. G. Zhao X. Walsh C. E. Gedeon J. Y. Cagle N. E.. (2015). Dorsal horn circuits for persistent mechanical pain. Neuron 87, 797–812. doi: 10.1016/j.neuron.2015.07.029, PMID: 26291162 PMC4562334

[ref82] Plummer N. W. Evsyukova I. Y. Robertson S. D. de Marchena J. Tucker C. J. Jensen P. (2015). Expanding the power of recombinase-based labeling to uncover cellular diversity. Development 142, 4385–4393. doi: 10.1242/dev.129981, PMID: 26586220 PMC4689223

[ref83] Ray R. S. Corcoran A. E. Brust R. D. Kim J. C. Richerson G. B. Nattie E.. (2011). Impaired respiratory and body temperature control upon acute serotonergic neuron inhibition. Science 333, 637–642. doi: 10.1126/science.1205295, PMID: 21798952 PMC3729433

[ref84] Raymond C. S. Soriano P. (2007). High-efficiency FLP and PhiC31 site-specific recombination in mammalian cells. PLoS One 2:e162. doi: 10.1371/journal.pone.0000162 17225864 PMC1764711

[ref85] Roth B. L. (2016). DREADDs for neuroscientists. Neuron 89, 683–694. doi: 10.1016/j.neuron.2016.01.040, PMID: 26889809 PMC4759656

[ref87] Sciolino N. R. Plummer N. W. Chen Y.-W. Alexander G. M. Robertson S. D. Dudek S. M.. (2016). Recombinase-dependent mouse lines for Chemogenetic activation of genetically defined cell types. Cell Rep. 15, 2563–2573. doi: 10.1016/j.celrep.2016.05.034, PMID: 27264177 PMC4915594

[ref88] Shi M.-Y. Ding L.-F. Guo Y.-H. Cheng Y.-X. Bi G.-Q. Lau P.-M. (2021). Long-range GABAergic projections from the nucleus of the solitary tract. Mol. Brain 14:38. doi: 10.1186/s13041-021-00751-4, PMID: 33608037 PMC7893933

[ref89] Sieber M. A. Storm R. Martinez-de-la-Torre M. Müller T. Wende H. Reuter K.. (2007). Lbx1 acts as a selector gene in the fate determination of somatosensory and viscerosensory relay neurons in the hindbrain. J. Neurosci. 27, 4902–4909. doi: 10.1523/JNEUROSCI.0717-07.2007 17475798 PMC6672097

[ref90] Singh P. Schimenti J. C. Bolcun-Filas E. (2015). A mouse Geneticist’s practical guide to CRISPR applications. Genetics 199, 1–15. doi: 10.1534/genetics.114.169771, PMID: 25271304 PMC4286675

[ref91] Sousa V. H. Miyoshi G. Hjerling-Leffler J. Karayannis T. Fishell G. (2009). Characterization of Nkx6-2-derived neocortical interneuron lineages. Cereb. Cortex 19, i1–i10. doi: 10.1093/cercor/bhp038, PMID: 19363146 PMC2693535

[ref92] Stirling L. C. Forlani G. Baker M. D. Wood J. N. Matthews E. A. Dickenson A. H.. (2005). Nociceptor-specific gene deletion using heterozygous NaV1.8-Cre recombinase mice. Pain 113, 27–36. doi: 10.1016/j.pain.2004.08.015, PMID: 15621361

[ref93] Takeoka A. Arber S. (2019). Functional local proprioceptive feedback circuits initiate and maintain locomotor recovery after spinal cord injury. Cell Rep. 27, 71–85.e3. doi: 10.1016/j.celrep.2019.03.010, PMID: 30943416

[ref94] Takeuchi T. Nomura T. Tsujita M. Suzuki M. Fuse T. Mori H.. (2002). Flp recombinase transgenic mice of C57BL/6 strain for conditional gene targeting. Biochem. Biophys. Res. Commun. 293, 953–957. doi: 10.1016/S0006-291X(02)00321-2, PMID: 12051751

[ref95] Tang J. C. Y. Rudolph S. Dhande O. S. Abraira V. E. Choi S. Lapan S. W.. (2015). Cell type–specific manipulation with GFP-dependent Cre recombinase. Nat. Neurosci. 18, 1334–1341. doi: 10.1038/nn.4081, PMID: 26258682 PMC4839275

[ref96] Todd A. J. (2010). Neuronal circuitry for pain processing in the dorsal horn. Nat. Rev. Neurosci. 11, 823–836. doi: 10.1038/nrn2947 21068766 PMC3277941

[ref97] van den Akker E. Forlani S. Chawengsaksophak K. de Graaff W. Beck F. Meyer B. I.. (2002). Cdx1 and Cdx2 have overlapping functions in anteroposterior patterning and posterior axis elongation. Development 129, 2181–2193. doi: 10.1242/dev.129.9.2181, PMID: 11959827

[ref98] van den Akker E. Reijnen M. Korving J. Brouwer A. Meijlink F. Deschamps J. (1999). Targeted inactivation of Hoxb8 affects survival of a spinal ganglion and causes aberrant limb reflexes. Mech. Dev. 89, 103–114. doi: 10.1016/s0925-4773(99)00212-9, PMID: 10559485

[ref99] Vrontou S. Wong A. M. Rau K. K. Koerber H. R. Anderson D. J. (2013). Genetic identification of C fibres that detect massage-like stroking of hairy skin in vivo. Nature 493, 669–673. doi: 10.1038/nature11810, PMID: 23364746 PMC3563425

[ref100] Wang Y. Wang F. Wang R. Zhao P. Xia Q. (2015). 2A self-cleaving peptide-based multi-gene expression system in the silkworm *Bombyx mori*. Sci. Rep. 5, 1–10. doi: 10.1038/srep16273 PMC4633692 26537835

[ref101] Watson J. A. Bhattacharyya B. J. Vaden J. H. Wilson J. A. Icyuz M. Howard A. D.. (2015). Motor and sensory deficits in the teetering mice result from mutation of the ESCRT component HGS. PLoS Genet. 11:e1005290. doi: 10.1371/journal.pgen.1005290, PMID: 26115514 PMC4482608

[ref102] Wiltschko A. B. Johnson M. J. Iurilli G. Peterson R. E. Katon J. M. Pashkovski S. L.. (2015). Mapping sub-second structure in mouse behavior. Neuron 88, 1121–1135. doi: 10.1016/j.neuron.2015.11.031, PMID: 26687221 PMC4708087

[ref103] Witschi R. Johansson T. Morscher G. Scheurer L. Deschamps J. Zeilhofer H. U. (2010). Hoxb8-Cre mice: a tool for brain-sparing conditional gene deletion. Genesis 48, 596–602. doi: 10.1002/dvg.20656, PMID: 20658520 PMC3566526

[ref104] Xue Y. Johnson R. DeSmet M. Snyder P. W. Fleet J. C. (2010). Generation of a transgenic mouse for colorectal Cancer research with intestinal Cre expression limited to the large intestine. Mol. Cancer Res. 8, 1095–1104. doi: 10.1158/1541-7786.mcr-10-0195, PMID: 20663863 PMC2923672

[ref105] Yamamoto M. Shook N. A. Kanisicak O. Yamamoto S. Wosczyna M. N. Camp J. R.. (2009). A multifunctional reporter mouse line for Cre- and FLP-dependent lineage analysis. Genesis 47, 107–114. doi: 10.1002/dvg.20474, PMID: 19165827 PMC8207679

[ref106] Yang S. Chang M. C. (2019). Chronic pain: structural and functional changes in brain structures and associated negative affective states. Int. J. Mol. Sci. 20. doi: 10.3390/ijms20133130, PMID: 31248061 PMC6650904

[ref107] Zhou L. Népote V. Rowley D. L. Levacher B. Zvara A. Santha M.. (2002). Murine peripherin gene sequences direct Cre recombinase expression to peripheral neurons in transgenic mice. FEBS Lett. 523, 68–72. doi: 10.1016/s0014-5793(02)02936-8, PMID: 12123806

[ref108] Zhu H. Aryal D. K. Olsen R. H. J. Urban D. J. Swearingen A. Forbes S.. (2016). Cre-dependent DREADD (designer receptors exclusively activated by designer drugs) mice. Genesis 54, 439–446. doi: 10.1002/dvg.22949 27194399 PMC4990490

