# Supplementary figures and images for: A new Hoxb8FlpO mouse line for intersectional approaches to dissect developmentally defined adult sensorimotor circuits
Source: Front Mol Neurosci. 2023 Aug 1;16:1176823. doi: 10.3389/fnmol.2023.1176823 (PMC10437123; doi:10.3389/fnmol.2023.1176823)

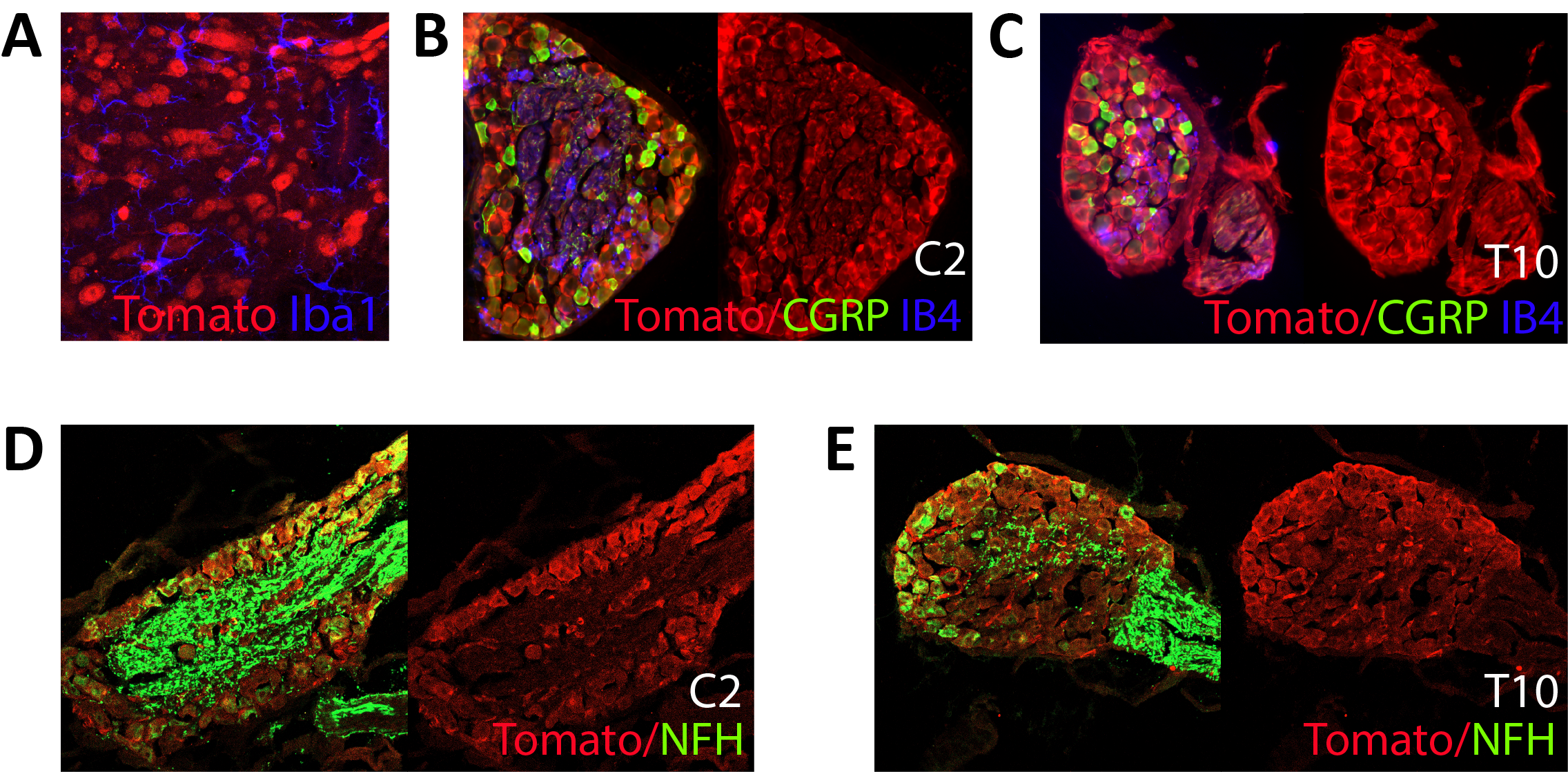

Supplement: SUPPLEMENTARY FIGURE S1 — Hoxb8FlpO expression is present in the DRG but not in neurons of the brain, Cdx2Cre expression can be observed in neurons of the brain. (A) Zoomed in image of a transverse lumbar spinal cord section from an adult Hoxb8FlpO;FSF-TdTomato mouse, showing Hoxb8FlpO expression is not present in spinal microglia. Hoxb8FlpO;FSF-TdTomato fluorescence (red) is not colocalized with Iba1+ microglia (blue). (B,C) Images of cryosectioned cervical (B) and thoracic (C) DRG from an adult Hoxb8FlpO;FSF-TdTomato mouse. Hoxb8FlpO;FSF-TdTomato fluorescence (red) is colocalized with the vast majority of DRG neurons, including CGRP+ (green) and IB4+ (blue) DRG neurons. (D,E) Images of cryosectioned cervical (D) and thoracic (E) DRG from an adult Hoxb8FlpO;FSF-TdTomato mouse. Hoxb8FlpO;FSF-TdTomato fluorescence (red) is colocalized with the large diameter DRG neuron marker NFH (green). [file Image_1.png]

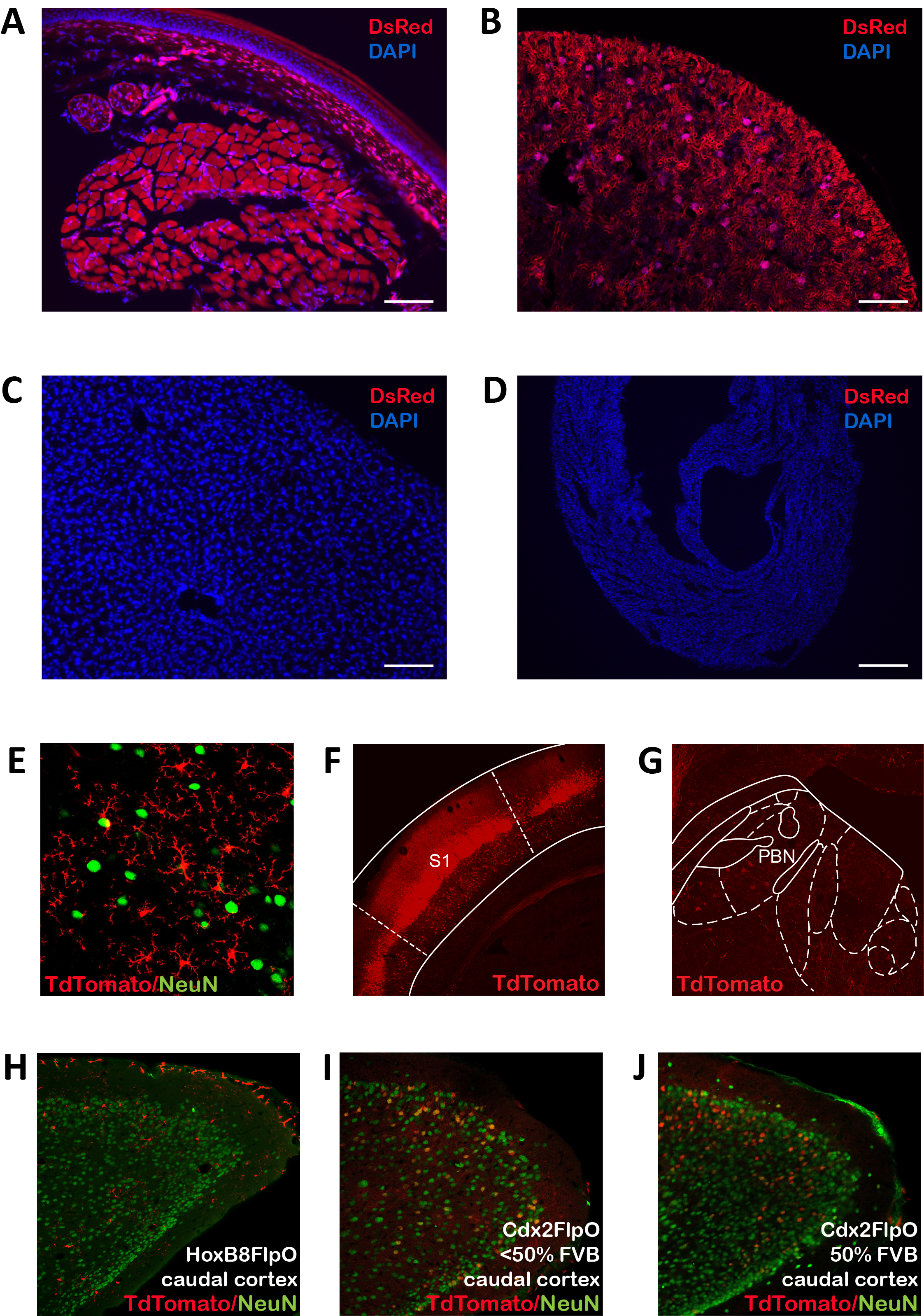

Supplement: SUPPLEMENTARY FIGURE S2 — Hoxb8FlpO expression is present in caudal viscera and brain microglia, but not in neurons of the brain. Cdx2Cre expression can be observed in neurons of the brain. (A) Image of cryosectioned hindpaw glabrous skin from an adult Hoxb8FlpO;FSF-TdTomato mouse, showing TdTomato expression (red) in the subcutis, with very sparse labeling in the reticular dermis. DAPI (blue) was used as a counterstain. (B) Image of cryosectioned kidney from an adult Hoxb8FlpO;FSF-TdTomato mouse, showing TdTomato expression (red) in endothelial cells. DAPI (blue) was used as a counterstain. (C) Image of cryosectioned liver from an adult Hoxb8FlpO;FSF-TdTomato mouse, showing an absence of TdTomato fluorescence in liver tissue. DAPI (blue) was used as a counterstain. (D) Image of cryosectioned heart from an adult Hoxb8FlpO;FSF-TdTomato mouse, showing an absence of TdTomato fluorescence in cardiac tissue. DAPI (blue) was used as a counterstain. (E) Zoomed in image of the cortex in coronal brain tissue from an adult Hoxb8FlpO;FSF-TdTomato mouse, showing the presence of TdTomato+ microglia (red) that are not colocalized with NeuN+ neurons (green). (F,G) Image of coronal brain and brainstem from adult Cdx2Cre;LSL-TdTomato mice, showing neuronal TdTomato fluorescence (red) in both the somatosensory cortex (F) and parabrachial nucleus (G) (H,I,J) Images of sagittal sections of caudal cortex from an adult Hoxb8FlpO;FSF-TdTomato mouse on a mixed genetic background (C57BL/6/FVB) (H) and from adult Cdx2NSE-FlpO; FSF-TdTomato mice on a 25% FVB background (I) and a 50% FVB background (J) showing TdTomato fluorescence (red) in neurons (NeuN in green) in the brain of Cdx2NSE-FlpO but not Hoxb8FlpO mice. We never observed brain neuronal expression in Hoxb8FlpO;FSF-TdTomato mice, independent of background. [file Image_2.png]

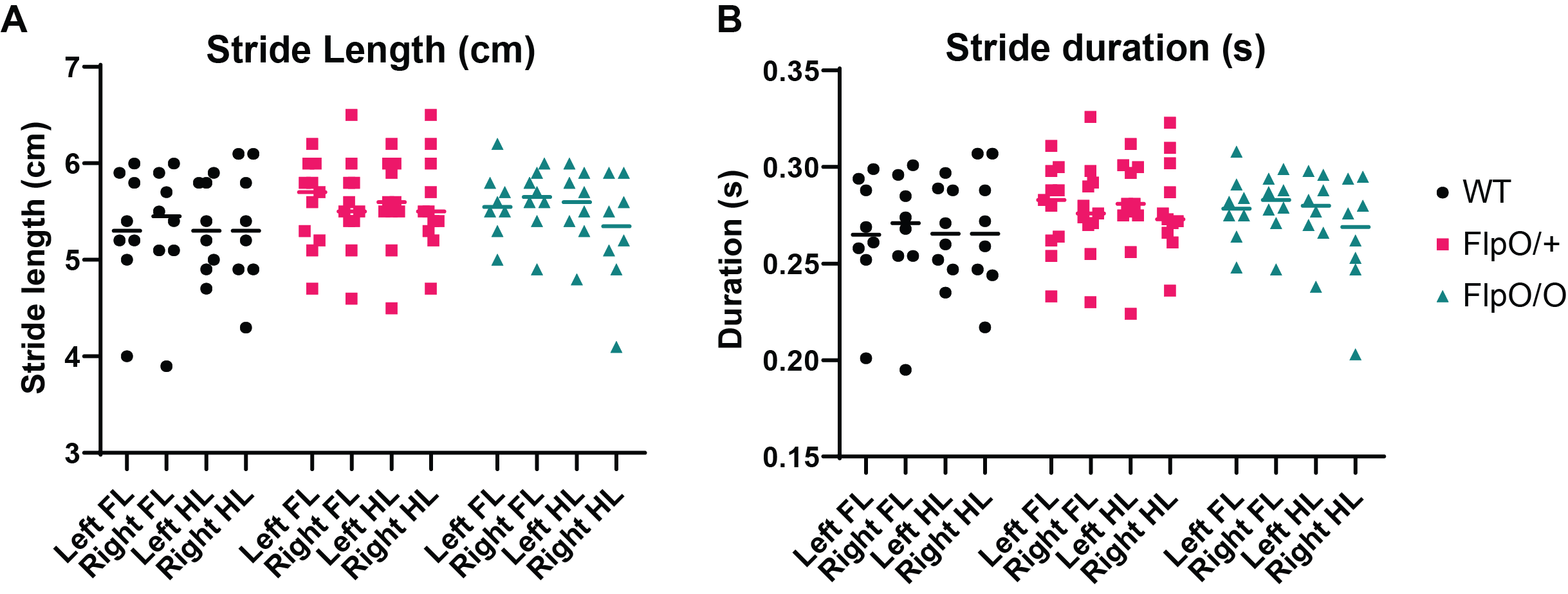

Supplement: SUPPLEMENTARY FIGURE S3 — Homozygous and heterozygous Hoxb8FlpO mice have normal locomotor gait (A) Hoxb8FlpO and Hoxb8FlpO/+ mice have normal stride length (cm) across all limbs compared to wild type controls. Data is represented as mean+individual data points (n = 9-15 mice per group), and significance was assessed using a one-way ANOVA with Tukey’s multiple comparisons. (B) Hoxb8FlpO and Hoxb8FlpO/+ mice have normal stride duration (s) across all limbs compared to wild type controls. Data is represented as mean+individual data points (n = 9-15 mice per group), and significance was assessed using a one-way ANOVA with Tukey’s multiple comparisons. [file Image_3.png]

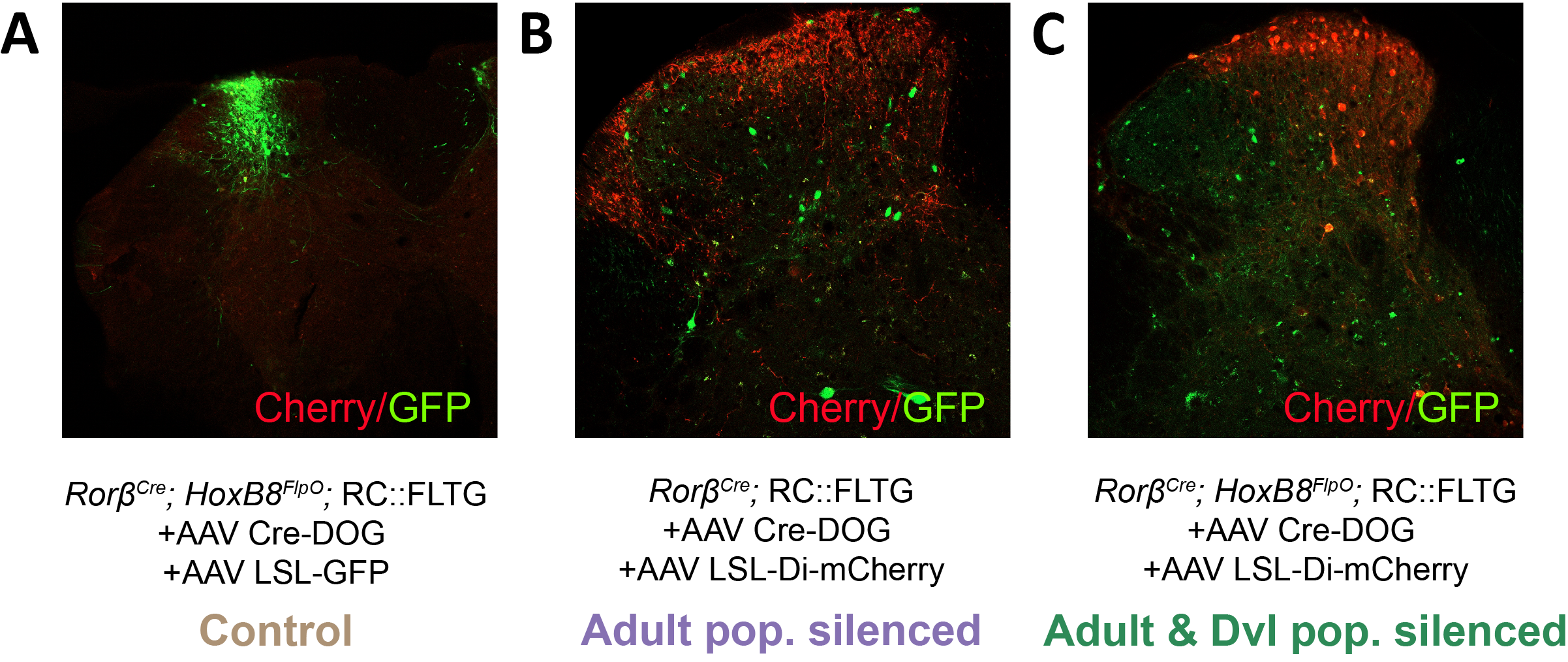

Supplement: SUPPLEMENTARY FIGURE S4 — The viral and transgenic intersectional strategy can target adult or adult & developmental Rorβ spinal neuronal populations. (A) Viral control: a Ror𝛽Cre;Hoxb8FlpO; RC::FLTG mouse was injected with Cre-DOG and AAV-LSL-GFP viruses. An image of a transverse section of the spinal cord shows Cre-DOG-dependent GFP expression at the site of injection. (B) Adult Rorβ spinal neuronal population silencing: a Ror𝛽Cre; RC::FLTG mouse was injected with Cre-DOG and AAV-LSL-Di-mCherry, targeting the adult Rorβ spinal neuronal population that still expresses Rorβ. An image of a transverse section of the spinal cord shows Cre-DOG-dependent Di-mCherry expression (red) in neural processes. (C) Adult & developmental Rorβ spinal neuronal population silencing: a Ror𝛽Cre;Hoxb8FlpO;RC::FLTG mouse was injected with Cre-DOG and AAV-LSL-Di-mCherry, targeting the adult & developmental Rorβ spinal neuronal population targeted by the Ror𝛽Cre;Hoxb8FlpO; RC::FLTG intersection. An image of a transverse section of the spinal cord shows Cre-DOG-dependent Di-mCherry expression (red). [file Image_4.png]
